# A cantilever-based, ultrahigh-vacuum, low-temperature scanning probe instrument for multidimensional scanning force microscopy

**DOI:** 10.3762/bjnano.13.95

**Published:** 2022-10-11

**Authors:** Hao Liu, Zuned Ahmed, Sasa Vranjkovic, Manfred Parschau, Andrada-Oana Mandru, Hans J Hug

**Affiliations:** 1 Empa, Swiss Federal Laboratories for Materials Science and Technology, CH-8600 Dübendorf, Switzerland.; 2 Department of Physics, University of Basel, CH-4056 Basel, Switzerland.

**Keywords:** atomic force microscopy, atomic resolution, instrumentation design, multimodal operation, ultrahigh vacuum

## Abstract

Cantilever-based atomic force microscopy (AFM) performed under ambient conditions has become an important tool to characterize new material systems as well as devices. Current instruments permit robust scanning over large areas, atomic-scale lateral resolution, and the characterization of various sample properties using multifrequency and multimodal AFM operation modes. Research of new quantum materials and devices, however, often requires low temperatures and ultrahigh vacuum (UHV) conditions and, more specifically, AFM instrumentation providing atomic resolution. For this, AFM instrumentation based on a tuning fork force sensor became increasingly popular. In comparison to microfabricated cantilevers, the more macroscopic tuning forks, however, lack sensitivity, which limits the measurement bandwidth. Moreover, multimodal and multifrequency techniques, such as those available in cantilever-based AFM carried out under ambient conditions, are challenging to implement. In this article, we describe a cantilever-based low-temperature UHV AFM setup that allows one to transfer the versatile AFM techniques developed for ambient conditions to UHV and low-temperature conditions. We demonstrate that such a cantilever-based AFM offers experimental flexibility by permitting multimodal or multifrequency operations with superior force derivative sensitivities and bandwidths. Our instrument has a sub-picometer gap stability and can simultaneously map not only vertical and lateral forces with atomic-scale resolution, but also perform rapid overview scans with the tip kept at larger tip–sample distances for robust imaging.

## Introduction

Atomic force microscopy (AFM) operated under vacuum or ultrahigh vacuum (UHV) conditions is beneficial for increasing measurement sensitivity, measuring samples at low temperatures [1], analyzing reactive surfaces [2], and studying atomic or molecular adsorbents with atomic or submolecular resolution [3]. The first AFM images with true atomic resolution were obtained by using cantilever-based AFM instruments, where cantilevers with stiffness of the order of few tens of newtons per meter were oscillated with amplitudes of a few nanometers [4–7]. Atomic resolution is achieved if the tip–sample distance is sufficiently reduced, such that short-ranged attractive or repulsive inter-atomic forces occur between tip apex atom and atoms at the surface. In recent years, functionalizing the tip apex with a lowly coordinated atom/molecule resulted in exceptional submolecular resolution at low temperature [8–11].

Tuning fork AFM has become increasingly popular for atomic resolution work performed under UHV conditions [12]. In tuning fork AFM, one of the prongs of the tuning fork is fixed to the tip holder, while the other one acts like a macroscopic cantilever. The comparatively large dimensions of the prongs facilitate the attachment of a small but macroscopic wire tip to the free prong. Compared to the typically used microscopic AFM cantilevers, the tuning fork sensor has a rather high stiffness, *k* ≈ 2 kN/m. This facilitates AFM operation with small oscillation amplitudes (*A <* 100 pm) because a snap-to-contact or instabilities of the phase-locked loop (PLL) driving the tuning fork oscillation do not occur. Furthermore, the tuning fork AFM does not require an extra deflection sensor such as the beam deflection or fiber-optical systems used for cantilever-based AFM, which substantially reduces instrumentation complexity. In fact, every existing scanning tunneling microscope (STM) can be transformed into a tuning fork-based AFM simply by replacing the rigid STM tip by a tuning fork with an attached tip and by adding an extra pre-amplifier and a PLL to drive the tuning fork oscillation and measure shifts in its resonance frequency arising from the tip–sample interaction. However, because of the macroscopic size of the tuning fork, the high stiffness of the sensor goes together with a low resonance frequency typically around 30 kHz. This substantially limits the minimally measurable tip–sample interaction force gradients such that very small AFM measurement bandwidths (typically below 10 Hz [13]) have to be used. These may be sufficient to record 2D images of a few square nanometers of scan size but will lead to extremely long measurement times for the acquisition of three-dimensional force volume maps (i.e., multiple 2D images). For example, the 3D frequency shift map acquired in the work of Albers et al. [14] with a volume of 1.6 × 0.8 × 0.12 nm^3^ and 256 × 119 × 61 pixels required a total acquisition time of 40 h, that is, it was measured with a pixel bandwidth of only 12.9 pixels per second.

While to date most atomic-resolution studies under UHV conditions are performed with tuning fork-based AFM, the vast majority of the AFM works performed under ambient conditions rely on microfabricated cantilevers that detect based on various mechanical properties and tips. Microfabricated cantilevers can be optimized for different AFM applications and operational environments. For AFM performed under ambient conditions, microfabricated cantilevers can, for example, be operated in different oscillation modes [15] or at multiple frequencies [16–23] to simultaneously map different sample properties. Further, the high resonance frequency of microfabricated cantilevers combined with high-bandwidth cantilever deflection detection permits video-rate scanning [24], real-time peak force detection [25], or a later artificial intelligence processing of the vast amounts of data acquired during imaging [26–27]. Under vacuum conditions, the resonance frequency-to-stiffness ratio of thin cantilevers proved to be beneficial for the measurement of ultrasmall forces [28] or, in combination with high cantilever quality factors, the detection of small magnetic fields [29]. For the latter, new tip–sample distance control operation modes were developed, which, again, relied on multifrequency techniques [30–32]. Such multimodal and multifrequency techniques have also been applied for AFM work performed under UHV conditions, for example, to measure atomic-scale forces in different special directions [33–35] or to work with sub-nanometer oscillation amplitudes for an improved detection of short-ranged inter-atomic forces [36–38].

Despite the success of AFM utilizing microfabricated cantilevers under ambient conditions, early work performed under UHV conditions, and high-sensitivity MFM under vacuum conditions, cantilever-based AFM may have lost the attention of the surface science and UHV AFM communities, possibly because of the ease of operation of tuning fork-based AFM and the availability of the corresponding instruments from various manufacturers. Here, we present the design of a robust and easy-to-use cantilever-based AFM instrument, which is not only optimized for atomic resolution work, but also permits high-bandwidth AFM operation and, thus, at least in principle, the implementation of more complex AFM operation modes (typically used for ambient environment AFM) also under UHV and low-temperature conditions. We further demonstrate that this instrument can be used for multimodal AFM operation, for example, to simultaneously map vertical and lateral forces and tunneling current signals with atomic resolution. It also permits the measurement of weak forces with high measurement bandwidths permitting the acquisition of overview images at larger tip–sample distances. Our instrument is thus well-suited to find specific locations in devices, map weak magnetic or electrostatic forces, and also permits the acquisition of smaller scan range atomic resolution images at specific locations.

This manuscript is organized as follows: The UHV and cryosystem are described in the next section, which is followed by a discussion of the main components of the AFM instrument and their functions in the section entitled “Microscope Design” and in various subsections to highlight technical and functional aspects of the various functional components of the AFM instrument.

A subsequent section entitled “Performance of the SPM” is dedicated to an analysis of the performance specifications of relevant AFM components such as its interferometric deflection sensor with subsections “Relevant AFM noise sources”, “Force gradient noise and measurement bandwidths”, and “STM noise spectrum and tip–sample gap stability measurements”.

Finally, various atomic-scale STM and AFM results described in section “Results and Discussion” structured into various subsections demonstrate the performance of our new AFM for such work. The last section entitled “Conclusion” finally summarizes the most relevant results and conclusions arising from our instrumentation approach and the presented experimental results.

## UHV Chambers and Cryosystem

**Figure 1:**
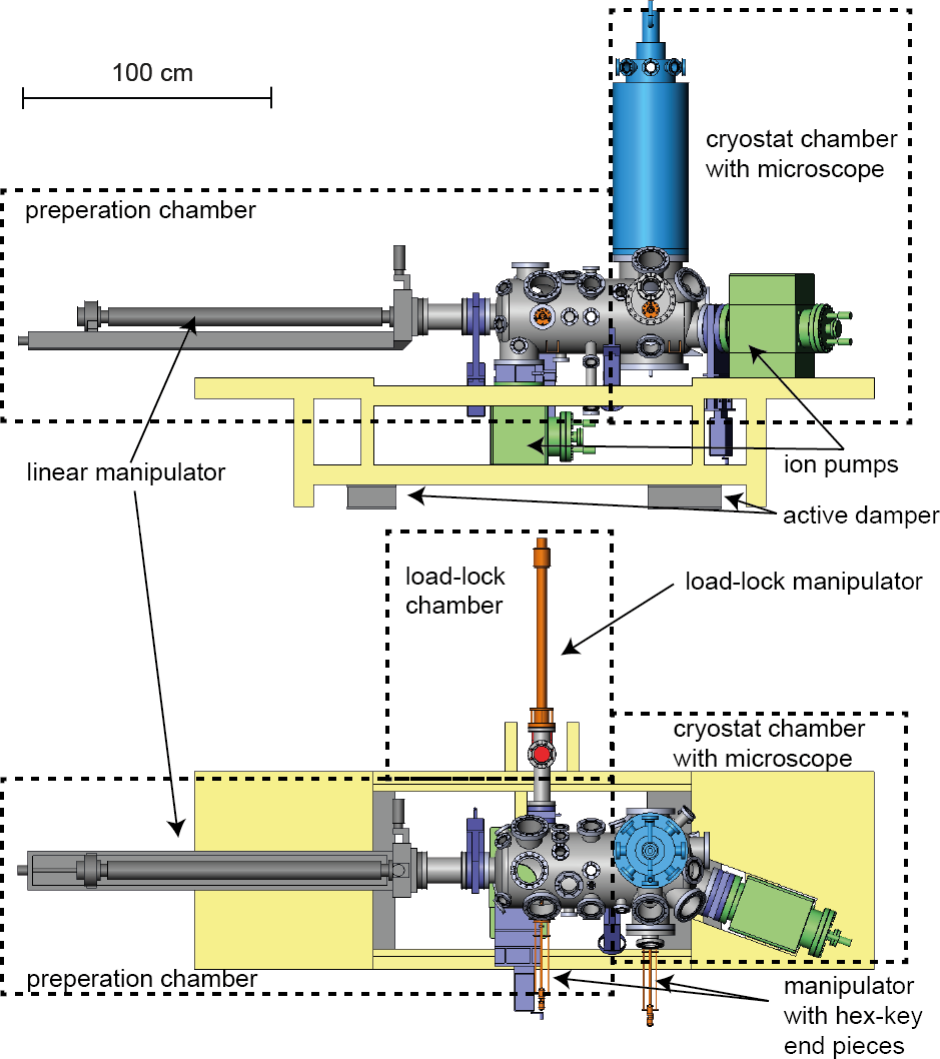
CAD drawings of the top and side views of the UHV system consisting of a cryostat chamber, a preparation chamber, and a load-lock chamber.

The UHV system [39] consists of a cryostat chamber and a preparation chamber with an attached load-lock as shown in Figure 1. The preparation chamber is equipped with various ports for the attachment of evaporators, a sputter gun, and surface science analytical tools. A rotatable coolable linear manipulator with two sample/cantilever receivers is used to transport sample and cantilever holders to the different positions of the preparation chamber and, finally, to transfer to the cryostat chamber. For the transfer of the sample/cantilever holders from the load-lock system to the linear manipulator inside the preparation chamber and, subsequently, from the linear manipulator to the corresponding receivers in the microscope, customized magnetic feedthrough manipulators with hex-key end-pieces are used.

Cryostat and preparation chamber are both pumped with 300 L/s ion pumps, which also include titanium sublimation sources. The load-lock chamber is pumped with a 67 L/s turbo pump. The bath cryostat manufactured by Cryovac [40] is mounted on top of the cryostat chamber outside the long axis of the chamber system (Figure 1); this permits a rapid transfer of (precooled) sample/cantilever holders from the manipulator to the microscope.

**Figure 2:**
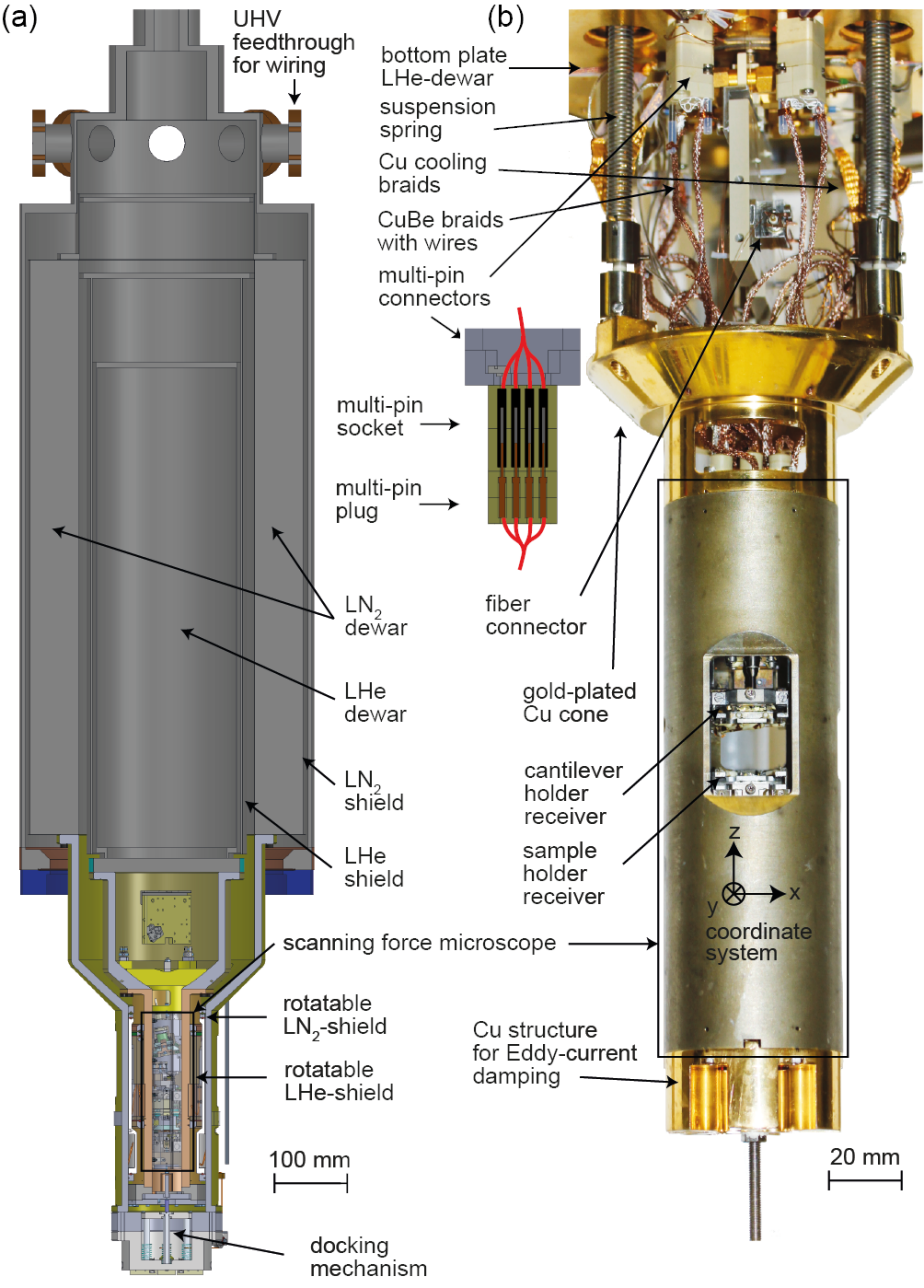
(a) The bath cryostat consists of two tanks, the inner tank holds 8 L of LHe and the outer tank holds 19 L of LN_2_, additionally with their own shields. The microscope is attached to the cone and hanging freely on three suspension springs as shown in the photograph (b).

The liquid helium (LHe) tank of the cryostat is surrounded by a liquid nitrogen (LN_2_) container and an additional heat shield, which is passively cooled by the evaporating He gases of the LHe tank (Figure 2a). The microscope is surrounded by two shields (a Au-plated oxygen-free high thermal conductivity (OFHC) copper LHe shield and an Al LN_2_ shield) of which there is an inner one mounted on the LHe cryostat bottom plate and an outer one connected to the bottom of the surrounding LN_2_ tank. With this construction, standby times of 80 h for the LHe tank and 96 h for the LN_2_ tank are achieved.

The scanning force microscope is attached to an OFHC Cu cone, hanging on three suspension springs that reach through cylindrical tubes running through the LHe tank and are mounted on top of the tank. Together with the Eddy current damping system mounted at the bottom of the cryostat, this provides excellent vibration isolation such that a tip–sample gap stability better than 1 pm can be obtained on a normal laboratory floor and with operation personnel in the same room. Note that all experiments discussed in section “Results and Discussion” have been carried out with personnel in the room.

The heat transfer between the microscope and the Cu bottom plate of the LHe of the cryostat is achieved through electrical connections between the microscope and the connectors on the cryostat bottom plate together with gold-coated Cu braids that connect the Cu cone to the cryostat bottom but keep a high mechanical flexibility (Figure 2b). Note that for the electrical connections between the connectors on the cryostat bottom plate and feedthroughs of the UHV system, low-heat-conductive phosphor bronze wires [41] are used. The wires run down along the LHe tank with several attachment points to further reduce the heat flow from the room-temperature UHV flange connectors to the cryostat bottom plate. For the Cu braids, in order to permit a defined grounding of the microscope independent of that of the UHV system, the Cu braids are electrically insulated through a sapphire plate from the cryostat bottom plate. For a more rapid cooling, the microscope can be pulled down by a LN_2_-cooled pulley system that locks in at the microscope bottom such that a mechanical contact between the Cu cone and the cone-shaped part of the LHe microscope shield is achieved.

To obtain access to the microscope, the LN_2_ shield can be rotated such that it connects to the inner LHe shield to open up an access window to the microscope for sample and cantilever holder transfer. The cantilever, the optical fiber, and the sample can be seen at a large optical viewing angle permitting a good microscopic view required for the positioning of the fiber relative to the cantilever. This allows, for example, for the positioning of the fiber end outside the long axis of the cantilever to measure torsional cantilever oscillation modes (see section “Performance of the SPM”) or the approach of the sample to the (cantilever) tip. An additional position of the shields opens a small access hole to the sample surface permitting the deposition of atoms or molecules on the cold sample.

## Microscope Design

We use a fiber-optical interferometer to measure the cantilever deflection. This deflection sensor type only requires placing the end of an optical fiber in close proximity to the cantilever. All electronic components remain outside the cryostat and the UHV system. Moreover, a fiber-optical interferometer sensor directly maps the cantilever deflection, whereas beam-deflection sensors only measure the angular change of the cantilever [42]. A fiber-optical interferometer, thus, permits a precise measurement of the cantilever oscillation amplitude, without the need of a complicated calibration [43–45]. Fiber-optical sensors can obtain sensitivities up to about 1 fm/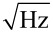 using Fabry–Pérot interferometry [46–47]. To date, however, we only implemented a simpler form of the interferometer composed of a cleaved and uncoated fiber end with a reflectivity of typically 4%. This limits the sensitivity of the interferometer to about 89 fm/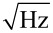, (see section “Results and Discussion” for the characterization of the interferometric deflection sensor).

**Figure 3:**
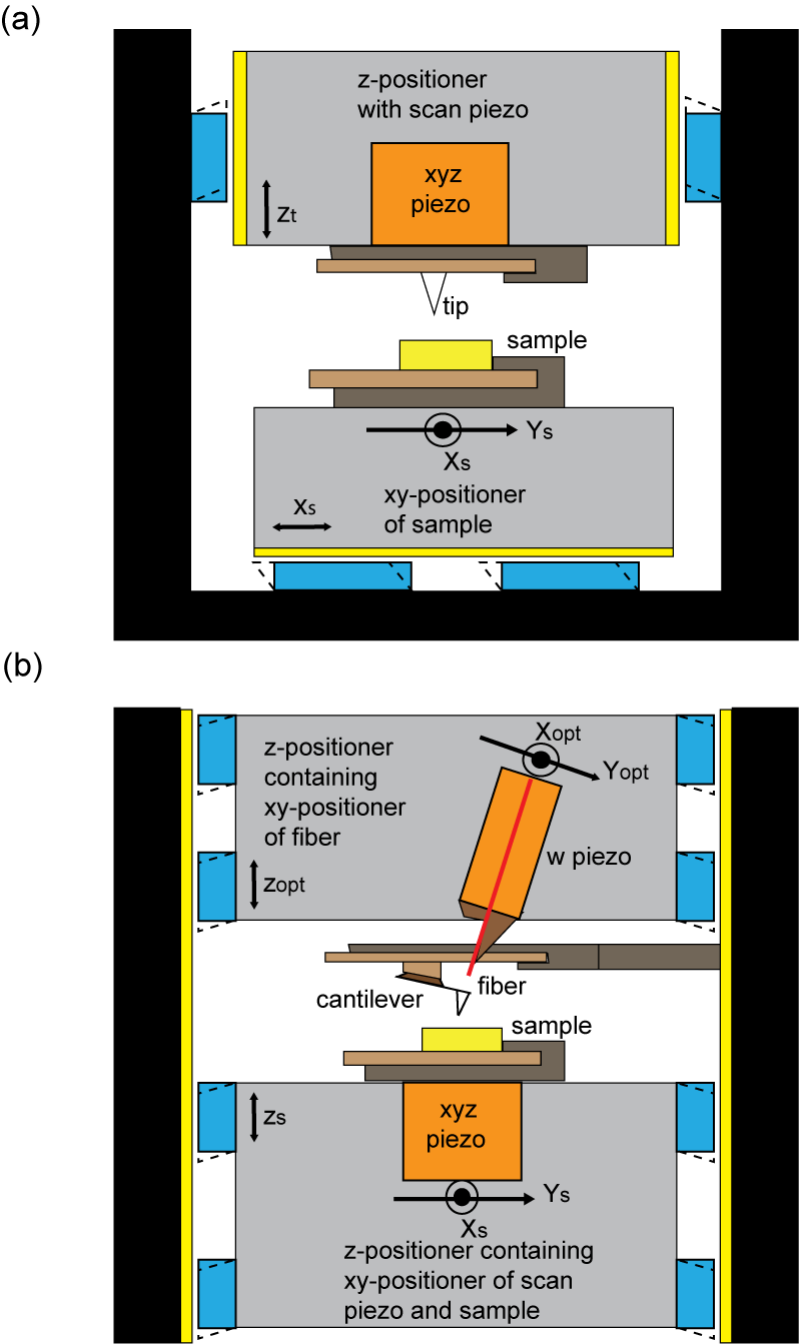
Schematics of the components of (a) a typical, classical STM/tuning fork-based AFM setup with the tip being scanned and (b) our AFM with the sample being scanned.

Figure 3a shows a typical setup for a UHV STM or tuning fork-based AFM. Preferably, the low-mass tip is scanned, while the heavier sample and sample receivers are mounted on a xy-positioning unit for the lateral positioning of the sample on a millimeter scale. To avoid stacking the z-positioning unit on top of the xy-positioning unit, the xyz-scan piezo and tip receiver unit are mounted inside a z-positioning unit, permitting the approach of the tip to the sample. Typically, shear piezo stacks are activated with a triangular voltage-versus-time signal to obtain a stick–slip motion of the slider of the positioning unit. In most instruments, the shear piezo stacks are mounted on the instrument body. For the z-positioning unit, typically, two sets of two piezo stacks are rigidly mounted (glued) to the instrument body, while a spring system is used to press the remaining two piezo stacks from the instrument body to the slider, permitting an adjustable clamping force [13]. The latter needs to be sufficiently large to obtain a good mechanical rigidity of the slider while still permitting a stick–slip motion of the slider.

In a cantilever-based AFM, the deflection sensor (here a cleaved fiber end) must be positioned relative to the cantilever. Scanning the cantilever tip would be impractical in this case, because it would require scanning the entire fiber positioning unit as well as the cantilever. Instead, the cantilever remains fixed to the instrument body, the fiber end is positioned on top of the cantilever, and the sample is scanned relative to the cantilever. This setup, however, requires stacking of the z-positioner on top of the xy-positioning unit or vice versa, making the design of a mechanically rigid instrument more challenging. In addition, the mass of the sample holder and sample holder receiver must be kept to a minimum in order to keep the resonance frequency of the xyz-scan piezo reasonably high, as required for a fast feedback. Furthermore, to avoid instrument downtime due to piezo tube fractures, sample exchange inside the UHV must be performed with minimal force applied to the scan piezo. The schematic setup of our instrument is displayed in Figure 3b. Our cantilever-based AFM instrument is made of two three-axis positioning modules that position (A) the sample relative to the cantilever tip, that is, the sample positioning unit, which is equipped with a sample scan-piezo), and (B) the fiber versus the cantilever back-surface, that is, the fiber positioning unit, which contains a piezo (w-piezo) for fine-tuning the fiber-to-cantilever distance and for keeping the interferometer at one of its most sensitive operating points.

### Sample positioning unit

For the sample positioning unit, Pan style positioners [48] are used. Triangular voltage pulse trains are applied to all shear piezo stacks simultaneously. In order to minimize the instrument volume and to maximize its mechanical rigidity, the scan piezo is integrated into the xy-positioning unit, which is contained inside the z-positioning unit, which moves inside the instrument body. Different to conventional z-positioning units as, for example, used in the work of Schwenk et al. [13] and Hug et al. [49], here the shear piezo stacks are attached to the sliding unit. This is one of the many design steps we have undertaken to improve the stability of the tip–sample gap. Because the shear piezos move together with the z-positioner containing the scan piezo with the sample, the mechanical loop from the tip to the sample becomes small in the approached state, whereas in the classical design (Figure 3a), the shear piezos are attached to the body of the instrument leading to the largest mechanical loop in the approached state.

**Figure 4:**
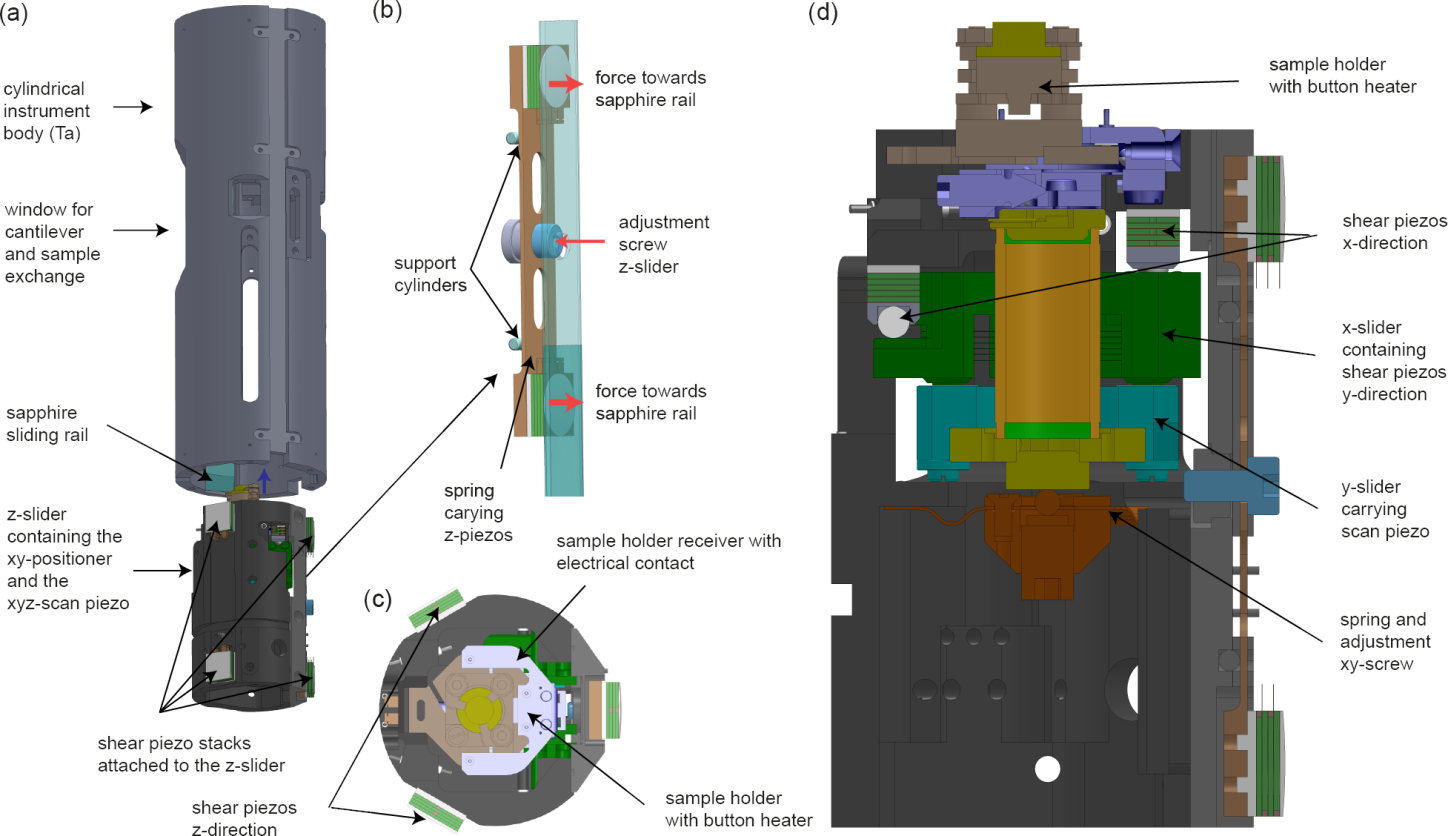
CAD drawings of the cylindrical body tube (a) and the leaf spring (b) carrying two of the total of six shear piezo stacks. The top and the cross-sectional views of the z-slider unit are shown in panels (c) and (d), respectively. The z-positioning unit also contains the xy-positioning unit and the xyz-scan tube carrying the sample holder receiver with the sample holder.

A further advantage of this design is that the instrument body can be manufactured as a single piece, in the form of a cylindrically shaped molybdenum tube (Figure 4a). As a result, only the sapphire plates, but not the piezo stacks, need to be glued on the inside walls of the body.

Our design with the piezos attached to the moving part, however, requires a spring system that applies a force from the inside towards the sapphire plates mounted on the inside of the instrument’s body tube (Figure 4a,b). Figure 4c,d shows the top and side views of the z-positioning unit containing the xy-positioning unit and the xyz-scan piezo carrying the sample receiver. While four of the six shear piezo stacks are glued to the z-slider unit, the two remaining stacks are glued to a leaf-spring assembly depicted in Figure 4a,b. The central screw (red arrow) pushes the leaf spring against two support cylinders, leading to an outward motion of the piezo stacks, pressing them against the sapphire rail (wide red arrows). With the screw in its released position, the sample z-positioning unit (and also that of the fiber, whichis not shown in Figure 4) can be placed inside the cylindrical body tube (blue arrow in Figure 4a) and the shear piezo stacks can be pressed towards the sapphire rails by tightening the adjustment screw, which is accessible through a hole in the cylindrical instrument body. Note that all piezo-motor adjustment screws are initially tightened under ambient conditions, that is, in air and at room temperature, to a level that the piezo motor slider still reliably moves at a piezo motor voltage pulse amplitude of ±70 V. The screws are then fixed with a small droplet of Torr Seal glue. The slider then still moves after a UHV system bake-out and at low temperatures at a piezo motor voltage pulse amplitude significantly smaller than ±270 V such that a reliable operation of the piezo motors is obtained.

To avoid any cross-coupling of the xy-motion as observed in earlier designs [49], two separated units with confined motions in the x- and y-directions are used here (Figure 4d). Such a stacking of two linear positioning units on top of the z-positioning units in a small building space, however, imposed various design challenges: First, a high mechanical rigidity must be obtained for a good tip–sample gap stability; second, the mechanical loop must be minimized and the design has to be kept as symmetrical as possible to reduce thermal drift; third, the design must allow for a precise adjustment of the pressure of the sliders towards the sapphire rails for the xy-directions.

**Figure 5:**
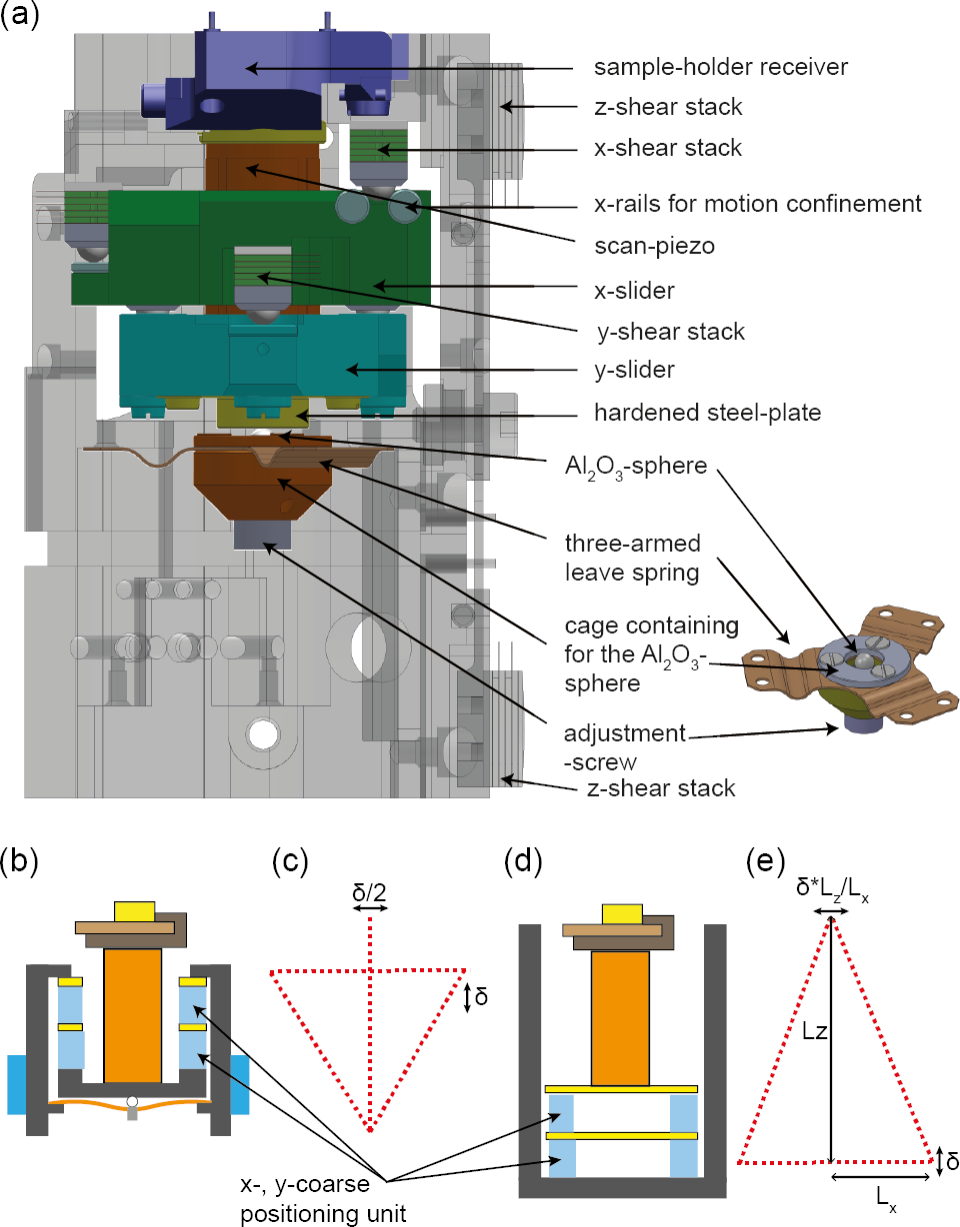
(a) CAD drawing of the z-positioning unit containing the xy-positioning units with scan piezo and, mounted to it, the sample holder receiver. (b) Schematic drawing of the assembly depicted in (a) highlighting the concentrical design and (c) the corresponding stability triangle. (d) Schematic drawing of a more conventional design, where the scan piezo is mounted on the top of the xy-positioning unit and (e) the corresponding stability triangle.

All these conditions can be fulfilled with a concentric design, where the shear stacks of the x-positioning unit are attached close to the top of the z-sliding unit (Figure 5a). The x- and y-sliders both use three shear piezo stacks and confine the motion along these directions by sliding an Al_2_O_3_ sphere attached to the shear stack inside a gap formed by two sapphire cylinders. The shear stacks for the x-direction are glued to the inside close to the top surface of the z-slider (Figure 5a). The x-slider is then arranged below these stacks and contains the three shear stacks of the y-direction, which then move the y-slider. The xyz-piezo is then attached to the top of the latter reaching through a hole in the x-slider to the top of the z-slider, such that the sample holder receiver is sufficiently high that the sample holder can be introduced into it. Both sliders are then pressed against their piezo stacks using a single three-armed leaf spring at the bottom with a sapphire sphere running on a hardened steel plate. Note that initially a sapphire plate was used. However, we found the plate cracked after a few days of piezo motor operation, presumably caused from an ultrasound-actuated contact resonance of the Al_2_O_3_ sphere on the sapphire plate arising from the triangular voltage signal applied during piezo motor operation. We found that replacing the sapphire plate by a mechanically more compliant hardened steel plate solved this issue. The sphere is contained in a cage mounted to a fine-thread, and a screw is used to adjust the force acting on the shear stacks of both the x- and y-sliders, facilitating the setting of a force sufficiently large to have a rigid assembly, but small enough to move the sliders at low temperatures, where the range of the shear stacks is significantly reduced.

With this concentric design, dimensional changes in the height of the shear stacks and sliders with temperature are at least partially compensated by those of the scan piezo. Together with the highly symmetric design along the x and y axes, this further reduces the thermal drift. Moreover, any mechanical excitation of the instrument, for example, from floor or acoustic vibrations may cause a wiggling motion of the slider of the size δ away from the supporting shear piezo stack (Figure 5b), which will translate into a later motion of δ/2 (Figure 5c). This is much smaller than the mechanically amplified motion of 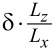 occuring in the classical stacked xy-motor design depicted in Figure 5d,e.

### Fiber positioning unit

**Figure 6:**
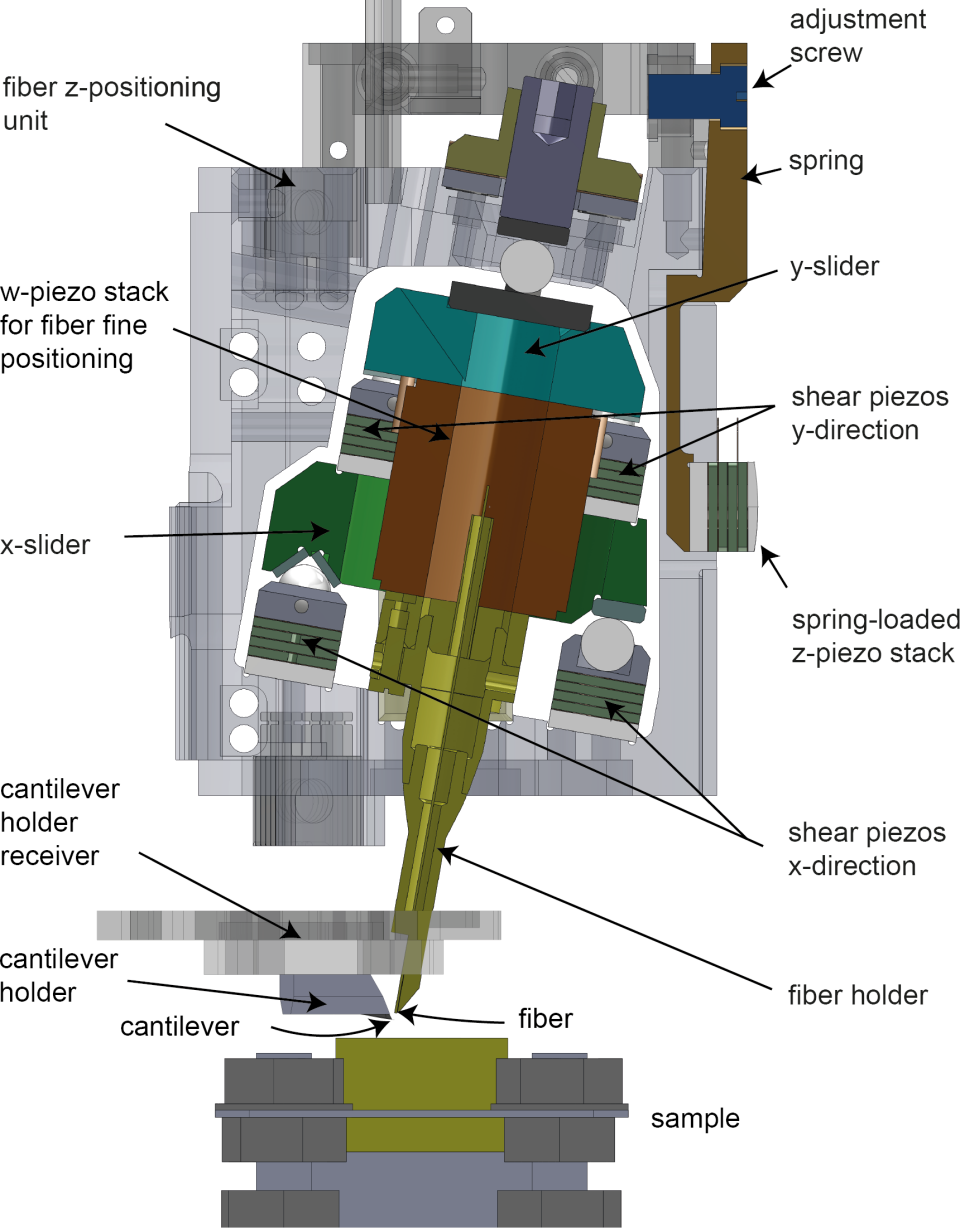
CAD sketch of the fiber z-positioning unit containing the x- and y-positioning unit. The assembly can be placed inside the cylindrical instrument body. After tightening the adjustment screw, the spring-loaded z-shear piezo stack and, consequently, the z-shear piezo stack attached to z-positioning unit will be pressed towards the sapphire rails on the inside of the cylindrical instrument body. The geometrical cantilever-to-fiber configuration is also highlighted. The cantilever and the fiber are tilted by 12° relative to the sample.

The same type of z- and xy-positioning units are also used to approach the fiber to the rear side of the cantilever and to position it along and perpendicular to the cantilever axis. Note that the xy-positioners for the fiber are tilted by the same 12° angle (Figure 6) as the cantilever to permit the y-positioning of the fiber parallel to the long axis of the cantilever. Similar to xy-positioners of the sample, the x- and y-positioners of the fiber can be independently adjusted without any cross-coupling. This permits a reliable positioning of the fiber either above the central axis of the cantilever or towards the cantilever edges to pick up torsional cantilever deflections (see section “Performance of the SPM”).

In order to maximize the sensitivity of the interferometric cantilever deflection measurement, a fiber-to-cantilever distance between two adjacent interference extrema must be selected and kept constant. This fine-positioning is performed by the w-piezo tube, which is implemented in the form of a stack of individual piezo plates (Figure 6).

### Sample and cantilever holders

**Figure 7:**
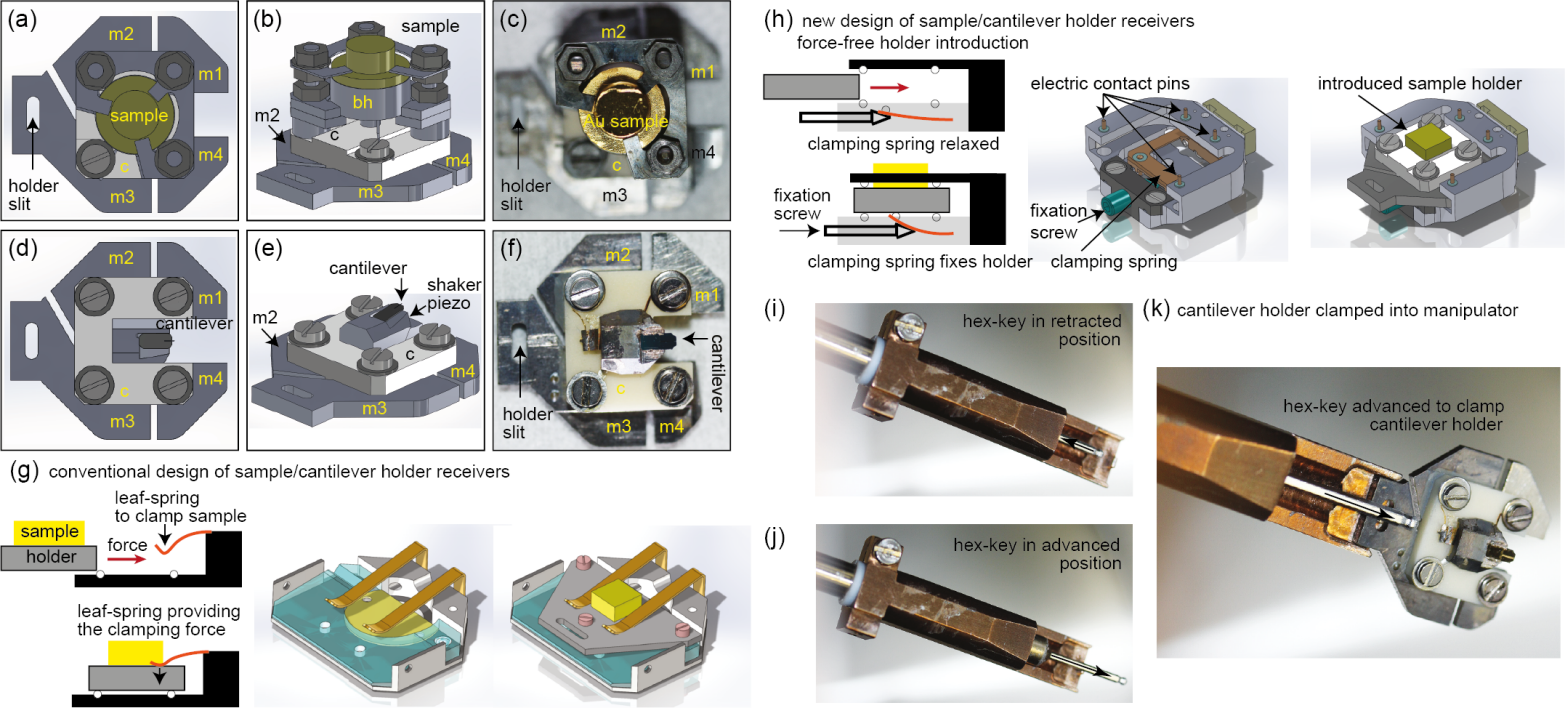
(a, b) Top and side view CAD sketches of a sample holder with a button heater for sample preparation. (c) A hat-shaped Au(111) single crystal mounted in a sample holder containing a button heater. (d, e) Top and side view CAD sketches of a cantilever holder with a shaker piezo integrated into the holder below the cantilever. (f) A cantilever holder with a mounted (glued) cantilever. The wire on the top right to the m1 contact plate is for the measurement of the tunneling current. The wire on the top left contacts the cantilever shaker piezo, while the wire on the bottom left provides the ground and shields the cantilever excitation voltage from the cantilever. (g) Typical sample/cantilever receiver design used in earlier instruments [45] where the sample/cantilever holder are clamped down by springs. (h) New sample/cantilever receiver design used here, permitting a force-free introduction/removal of the sample/cantilever from the corresponding receiver. (i, j) Manipulator with a rotatory hex-key end piece that can be moved along its long axis to clamp a sample/cantilever holder for a safe transport between the chamber transport system and the sample/cantilever holder receiver in the AFM (k).

UHV AFM instrumentation typically permits the in situ exchange of samples and (cantilever) tips. For this, sample and cantilever are mounted on corresponding holders (Figure 7a–c and Figure 7d–f, respectively). For efficient UHV AFM experimental work, it is favorable to have a conveniently large number of different sample and cantilever holders. Such holders with electrical contacts, however, are complex, and their fabrication and assembly typically require considerable efforts. For this reason, all our sample/cantilever holders use the same four laser-cut metal parts as base plates (m1–m4) connected via a simple ceramic center piece (Figure 7f), on top of which different assemblies can be arranged, for example, to carry a sample button heater (Figure 7a–c) or a shaker piezo for the mechanical excitation of the cantilever oscillation (Figure 7d–f).

### Sample and cantilever receivers

These sample/cantilever holders can be transported through the UHV system using the linear manipulator. In most instruments, the receivers for the sample or cantilever holders use clamping springs to fix the holders in their positions (Figure 7g). However, the introduction of the sample/cantilever holder into the corresponding receiver requires overcoming frictional forces, which may lead to a deformation of the holding springs and, consequently, to a loose fixation of the sample/cantilever holder in its receiver. Moreover, the sliding motion will also create wear particles, which may contaminate the surface of the sample or the inside of the instrument. Generally, such receiver designs compromise between a sufficiently large clamping force and the frictional forces that need to be overcome to exchange the sample/cantilever.

Here, we designed a new type of sample/cantilever receivers containing an adjustable clamping spring to overcome these inherent problems (Figure 7h). When the sample/cantilever holder is introduced or removed from the receiver, the clamping spring is in a lower position, not touching the sample/cantilever holder, such that the latter can be introduced or moved without applying forces to the receiver. The fixation of the sample/cantilever holder is then performed by rotating the fixation screw, which pushes the clamping spring against the sample/cantilever holder (Figure 7h). The required rotary motion can be applied via a customized magnetic-feedthrough manipulator, which includes a rotatable hex-key end piece (Figure 7i,j). This end piece can further be moved along its axis, permitting the clamping of a sample/cantilever holder and, thus, allowing its safe and rapid transport between the linear manipulator head and the corresponding receivers in the AFM (Figure 7k).

Note that we have tested different designs for the screw-activated clamping mechanism. We found the mechanism to be reliable (permits operation for more than a year with lots of sample/cantilever holder exchanges) with a conical screw coated by tungsten disulfide running in a thread of the receiver (fixation screw and thread piece in Figure 7h). The screw or the part with the thread can easily be replaced in the case of extensive wear. The conical end of the screw then presses on a sapphire inlay glued to the bottom part of the clamping spring.

The fixation of the sample/cantilever holder inside the corresponding receiver also leads to an electrical contact between pads on the sample/cantilever holder and contact pins on the receiver. We typically use three (out of the four) contact pins on the holder top, but can also use two contact pins on the clamping springs and, hence, have a total of five electrical contacts. Because four top contacts overdefine the plane of the sample/cantilever holder, the holder typically has a smaller thickness in one of the front contact areas, such that only one of the front electrical pins makes contact with the holder. A modified design of our holder with more (spring-loaded) electrical contacts from the top has been recently described by Schwenk and co-workers [13].

The sample holder receiver, which is fixed to the top of the scan piezo tube is manufactured from DISPAL Aluminum 225 [50] to reduce its mass. For the cantilever receiver, which is not scanned but directly mounted to the tubular Mo body of the instrument, Mo is used.

### Modular wiring design

In order to facilitate instrument service, modification, or repair, every module of the microscope has a separate wiring branch and can thus be easily removed from the microscope without having to remove wires or connectors from the module.

For the sensitive signal inputs and outputs, such as STM current and sample bias voltage, coaxial cables Lakeshore CC-SS-100 [51] with a SMA connector at their ends are used. These are wired to the two front electrical contact pins (Figure 7d–f). For all other contacts and also the wiring for the scan piezo, piezo motors, piezo for the mechanical actuation of the cantilever oscillation, temperature sensor (below the sample holder), and heaters, silver-coated Cu wires (DABURN 2451 [52]) are used. For electrical screening, wires carrying opposite voltages (X+ and X−, Y+ and Y− for the scanner as well as W+ and W− for the w-piezo) are twisted. Furthermore, groups of twisted pairs are contained in a CuBe braid with a custom-built multi-pin plug at the end, which is then plugged into the corresponding socket on the bottom plate of the LHe tank of the cryostat (Figure 2b).

From the multi-pin socket at the cryostat bottom, the wire bundles for specific instrument modules are reordered into functional groups, for example, one group containing all wires for the piezo positioners, for sample scan and w-piezo, for electrical contacts to the sample and cantilever, and for instrument heaters and temperature sensors.

### Interferometer system

**Figure 8:**
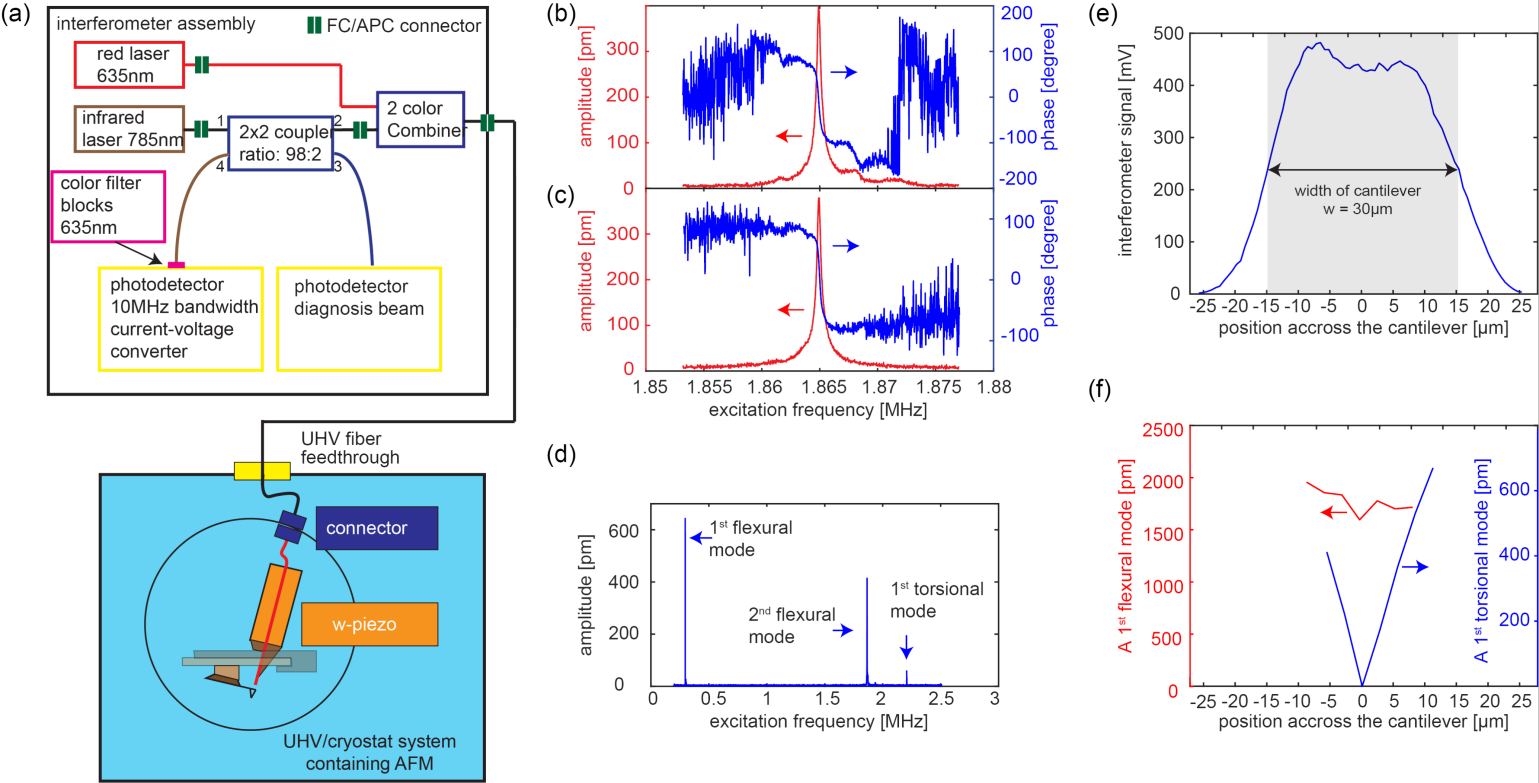
(a) Setup of the interferometer system. (b, c) Amplitude and phase as a function of the frequency for mechanical and optical cantilever excitation, respectively. (d) Wide frequency range mechanical excitation spectrum of the cantilever showing the first and second flexural and first torsional resonances. (e) Interferometer signal as a function of the fiber position across the cantilever (displayed schematically by the gray area). (f) Measured oscillation amplitudes of the cantilever for the first flexural (red) and first torsional oscillation modes (blue), respectively. The torsional oscillation modes vanish if the fiber is positioned above the central axis of the cantilever.

The layout of the fiber-optical interferometer system is depicted in Figure 8a. A similar interferometer system has been developed by Miyahara and co-workers [53]. To perform the interferometry, we use a Sony SLD201 V3 laser diode with a wavelength of 785 nm coupled via an optical insulator to a Au-coated monomode optical fiber having a core diameter of 5 μm [54] delivering a maximum of 9.3 mW into the fiber at a drive current of 140 mA. To keep the temperature of the laser diode constant, it is mounted onto a Thorlabs TCLDM9 [55] thermoelectric cooler block, and the laser diode is operated at constant current. A combined laser diode and temperature controller (Thorlabs ITC502 [55]) controls both the current and the temperature. In contrast to earlier designs, which relied on a 50:50 fiber-optical 2 × 2 coupler, the increased power of the laser diode permits [43–44] the use of a 98:2 fiber-optical 2 × 2 coupler with the laser diode connected to one of the two 2% branches. Thus, for the 9.3 mW maximum input power, only 1.4%, that is, 127 μW reaches the fiber end in the AFM, because of additional losses in the optical connectors. This minimizes the light coupled to UHV/cryostat system (blue shaded area in Figure 8a) containing the AFM and, thus, a potential heating effect. It also maximizes the intensity of the light reflected back from the fiber-end/cantilever assembly to the measurement photodiode, which leads to about 50 μW on the measurement photodiode, which is part of a 10 MHz bandwidth current-to-voltage converter.

The interferometer system can be equipped with an additional laser diode (LP633-SF50 [55]) with a wavelength of 635 nm coupled into the fiber with a 2-color-combiner (NR73A1 [55]), allowing for an optical excitation of the cantilever oscillation. We found that a mechanical excitation of the higher cantilever oscillation modes can become challenging when other resonances arising from the mechanical setup of the cantilever holder with its excitation piezo are located close to the cantilever resonance. Figure 8b,c shows the measured amplitude and phase of the second flexural cantilever resonance excited mechanically (by the shaker piezo on the cantilever holder) or optically (using a DC and AC current for the 635 nm laser diode to oscillate its light intensity), respectively. Note that the additional color filter placed in front of the photodiode prevents the backreflected 635 nm light to reach the photodiode, such that only the interference of the 785 nm laser light is used to map the cantilever deflection. For the specific cantilever, the dependence of amplitude and phase on the excitation frequency expected for a harmonic oscillator becomes disturbed significantly by a nearby mechanical resonance of the cantilever holder for a mechanical excitation of the cantilever (Figure 8b). Because the cantilever resonance frequency changes when the cantilever interacts with the surface, that is, in AFM operation mode, the 180° phase shift from the cantilever resonance can overlap with the phase shift arising from the mechanical resonance, leading to a failure of the phase-locked loop to track the cantilever’s resonance frequency [53,56]. In such a case, optical excitation is preferred. In contrast to the mechanically excited cantilever (Figure 8b), an optical excitation (Figure 8c) leads to clean harmonic oscillator-like phase and amplitude versus frequency curves.

Note that the 10 MHz bandwidth of the photodiode current-to-voltage converter permits the measurement of higher flexural and torsional modes occurring at frequencies well beyond 1 MHz (Figure 8d). To measure torsional cantilever oscillation modes, the fiber needs to be positioned outside the long cantilever axis, close to the boundary of the cantilever [57]. Figure 8e shows the measured interferometer signal as a function of the fiber position across the cantilever. For a cantilever width *w* of 30 μm, we can estimate the laser spot size to be about 10–15 μm on the cantilever. Figure 8f shows the measured size of the first flexural (red curve, left vertical axis) and torsional (blue curve and right vertical axis) cantilever oscillation mode with frequencies of 2.959 kHz and 2.206 MHz as a function of the position of the fiber across the cantilever. While the flexural mode oscillation signal (red curve in Figure 8f) remains roughly constant (with a slight dip in the middle of the cantilever similar to that observed in the interference signal from Figure 8e), the torsional mode signal vanishes at the center of the cantilever (blue curve in Figure 8f). The absence of the signal at the center of the cantilever can also serve as a signature to clearly identify a torsional oscillation mode.

## Performance of the SPM

### Relevant AFM noise sources

Compared to tuning fork sensors, microfabricated low-mass cantilevers offer considerable advantages concerning measurement noise and measurement bandwidth. They further permit multimodal AFM operation schemes [58] at the cost of an increased complexity of the instrumentation, arising from the need of an additional deflection sensor, which needs to be positioned relative to the cantilever. As discussed by Kobayashi et al. [59], the measurement noise arises from three different noise sources, that is, thermal noise of the cantilever (thermal noise), noise of the deflection sensor (deflection noise) and noise arising from fluctuations of the oscillator circuitry driving the cantilever oscillation (oscillator noise). These noise sources all limit the minimally measurable rms z-derivative of the z-component of the force, as given by the expressions:


[1] $${\frac{\partial F_{z}}{\partial z}|}_{\text{th}}=\frac{1}{A_{\text{rms,}i}}\cdot \sqrt{\frac{4k_{\text{B}}Tk_{i}B}{2\pi f_{i}Q_{i}}}\propto \sqrt{\frac{k_{i}}{f_{i}Q_{i}}},$$

[2] $${\frac{\partial F_{z}}{\partial z}|}_{\text{def}}=\frac{1}{A_{\text{rms,}i}}\cdot \frac{n_{\text{eq}}2k_{i}B^{\frac{3}{2}}}{\sqrt{3}f_{i}}\propto \frac{k_{i}}{f_{i}},$$

[3] $${\frac{\partial F_{z}}{\partial z}|}_{\text{osc}}=\frac{1}{A_{\text{rms,}i}}\cdot \frac{n_{\text{eq}}k_{i}\sqrt{B}}{Q_{i}}\propto \frac{k_{i}}{Q_{i}},$$

where *k_i_*, *f_i_*, *Q_i_*, and *A*_rms_*_,i_* are the stiffness, free resonance frequency, quality factor, and rms oscillation amplitude of the *i*-th cantilever oscillation mode (different flexural or torsional oscillation modes), respectively; *k*_B_ = 1.38 × 10^−23^ J·K^−1^ is the Boltzmann constant, *T* is the temperature, *B* is the bandwidth at which the measurement is performed, and *n*_eq_ is the noise of the deflection sensor, given in units of m/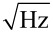. The minimally measurable rms z-derivative of the z-component of the force then arises from the sum of all noise sources and is thus given by:


[4] $${\frac{\partial F_{z}}{\partial z}|}_{\text{tot}}=\sqrt{\sum_{i=\text{th,def,osc}}{\frac{\partial F_{z}}{\partial z}|}_{i}^{2}}.$$

For rectangular cantilevers, the flexural modal stiffness and resonance frequency of the *i*-th flexural oscillation modes are related to the first flexural mode stiffness and resonance frequency, respectively, by:


[5] $$k_{i}=k_{1}\cdot {\left[\frac{\alpha_{i}}{\alpha_{1}}\right]}^{4},$$

[6] $$f_{i}=f_{1}\cdot {\left[\frac{\alpha_{i}}{\alpha_{1}}\right]}^{2},$$

where α*_i_* = {1.8750, 4.6941, 7.8548, … } are coefficients defined by the characteristic equation of an oscillating rectangular cantilever with one free end [60]. Note that for a typical non-contact AFM experiment, the tip end of the cantilever can be considered as free because the cantilever force constant is generally much smaller than the measured derivative of the tip–sample interaction force [61]. The force constant of a rectangular cantilever and its first flexural mode stiffness, respectively, are given by:


[7] $$c_{\text{L}}=\frac{E_{\text{Si}}t^{3}w}{4L^{3}}\text{ and }k_{1}=\frac{c_{\text{L}}\alpha_{1}^{4}}{12},$$

where ρ_Si_ = 2331 kg/m^3^ and *E*_Si_ = 1.69 × 10^11^ N/m^2^ are the density and the elastic modulus of silicon, respectively; *L*, *w*, and *t* are the length, width, and thickness of the cantilever, respectively. While the first two geometrical dimensions are well-defined by the fabrication process and can easily be measured by electron microscopy, the thickness *t* of the cantilever is best obtained from the measured first mode flexural resonance frequency *f*_1_ using:


[8] $$t=\frac{2\pi f_{i}L^{2}}{\alpha_{1}^{2}}\cdot \sqrt{\frac{12\rho_{\text{Si}}}{E_{\text{Si}}}}.$$

The expressions for the minimally measurable force derivative (Equation 1 and Equation 2) arising from thermal and deflection sensor noise, respectively, reveal that a high quality factor (for a low thermal noise) and a low modal stiffness resonance frequency ratio (for both noise sources) are beneficial for a high signal-to-noise ratio or large measurement bandwidths. Because the stiffness depends on 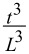 (Equation 7), whereas the resonance frequency is proportional to 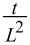 (as derived from Equation 8), a low stiffness-to-frequency ratio at a reasonably high resonance (several tens or hundreds of kilohertz) is best obtained with microfabricated thin cantilevers. A low cantilever thickness is further beneficial for the support loss quality factor (which is one of the relevant energy loss terms describing different mechanisms responsible for the loss of energy from a specific cantilever oscillation mode), because *Q*_support_ ∝ 1/*t*^3^ [62].

The measurement of magnetic, electric, or van der Waals forces is, thus, best done with thin cantilevers. These cantilevers typically have resonance frequencies of a few tens of kilohertz (comparable to that of a tuning fork) but a stiffness that is about four orders of magnitude smaller than that of a tuning fork, resulting in a reduction of the thermal and deflection noise by, respectively, two and four orders of magnitude (see Table 1) assuming the same quality factor. Note that, for a soft cantilever, the deflection noise obtained with typical deflection sensors is negligible such that thermal noise is dominant. Recently, Feng et al. [29] have demonstrated that at room temperature a force derivative of 78 nN/m is detectable in a bandwidth of 1 Hz, which is of particular importance for the measurement of small magnetic forces and for MFM with optimized lateral resolution.

To obtain atomic resolution, cantilevers with a higher stiffness are required to meet the stability criteria:


[9] $$c_{\text{L}}>-{\frac{\partial F_{\text{ts}}}{\partial z}|}_{\max},$$

or


[10] $$c_{\text{L}}\cdot A>{\left|F_{\text{ts}}\right|}_{\max},$$

where *F*_ts_ is the tip–sample interaction force. From Equation 9, the cantilever stiffness must surpass the highest attractive force gradient acting on the cantilever to prevent a snap to contact. Alternatively, such a snap-to-contact can also be prevented by a sufficiently large cantilever oscillation amplitude, such that the restoring force surpasses the maximum attractive force (Equation 10). Further, sufficient energy must be stored in the cantilever oscillation such that stochastic energy loss events caused by stochastic position changes of the tip apex [63] or sample atoms in interaction with the tip will not unlock (crash) the phase-locked loop. To obtain an oscillation energy of a few tens of electronvolts at smaller cantilever oscillation amplitudes of, for example, *A* = 100 pm, typically force constants of a few hundred newtons per meter are required. This permits a stable oscillation of the cantilever and tracking of the resonance frequency shifts, even in the presence of energy loss processes arising from stochastic changes of atomic positions at the tip apex or sample atoms interacting with the tip [64]. Such stiffnesses are typically obtained in the second flexural oscillation mode of cantilevers with a first flexural mode stiffness of a few tens of newtons per meter (Equation 5). While the second modal stiffness of such a cantilever has about the same order of magnitude as that of a tuning fork, its resonance frequency is almost two orders of magnitude higher. According to Equation 1 and Equation 2, cantilever sensors have thermal and deflection noise advantages of, respectively, about one and two orders of magnitude under the assumption that the quality factor and noise of the deflection sensor can be compared to those of a tuning fork. Moreover, the deflection noise (Equation 2) depends on the 1.5-th power of the bandwidth, whereas the thermal noise (Equation 1) depends on the square root of the measurement bandwidth. For a hard cantilever and, likewise, for a tuning fork sensor, the deflection noise can become the dominant noise source, such that a low stiffness-to-resonance frequency ratio becomes particularly relevant.

Note that the oscillator noise (Equation 3) depends on the noise of the force sensor *n*_eq_, the cantilever stiffness *k_i_*, and the quality factor *Q_i_*, but not on the resonance frequency *f_i_* of the cantilever. Hence, different from the thermal and detector noise terms, having a high resonance frequency is not beneficial. However, as Kobayashi already pointed out [59], the oscillator noise is not relevant for a high-Q cantilever, provided that the thermal noise peak is sufficiently larger than the noise of the deflection sensor, that is, the thermal noise amplitude at the corner frequencies, 
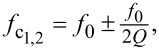
 is considerably larger than the background noise of the deflection sensor. This is typically fulfilled for the first and second flexural and first torsional oscillation modes of microfabricated cantilevers such that the oscillator noise contribution is negligible. Table 1 summarizes the stiffness-to-frequency ratios for typical microfabricated cantilevers and tuning forks. According to Equation 1 and Equation 2, these ratios determine the minimally measurable force derivative or for the obtainable measurement bandwidth (measurement speed).

**Table 1:** Thermal and detector noise sensitivities of different cantilevers and oscillation modes normalized to that of a tuning fork operated at the same temperature (TF in the table) sensor (higher numbers, i.e., higher measurement sensitivities are better). Line 1: High-quality factor MFM cantilever operated under vacuum conditions [29] in its first flexural mode. Lines 2 and 3: Typical cantilever used for atomic resolution work, operated in the first and second flexural mode, respectively. Line 4: Tuning fork sensor [13] operated in its flexural mode for comparison with lines 1–3. Line 5: For bi-axial force gradient measurements with a tuning fork [65], its length extension mode was used to map the vertical force gradient. Line 6: The cantilever with the properties given in line 3 now compared to the sensitivity of the tuning fork length extension mode given in line 5. Line 7: Lateral force sensitivity obtained with the first torsional oscillation mode of a cantilever (that can be measured simultaneously with its second flexural mode, line 3), which needs to be compared to the sensitivity of the tuning fork operated in its conventional flexural mode (line 4).

		*k*	*f*_0_	*Q*	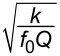	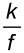
		[N/m]	[kHz]	[k]	[normalized]	[normalized]

measurement of vertical force gradient						

1	MFM 1st flex	0.5	50	250	129.10	6667
2	AFM 1st flex	25	300	100	28.29	800
3	AFM 2nd flex	982	1,880	10	3.57	127
4	TF flex	2,000	30	100	1	1

simultaneous measurement of vertical and lateral force gradients						

5	TF l.ext	1.43M	567,000	N/A	N/A	1
6	AFM 2nd flex	982	1,880	10	N/A	4831
7	AFM 1st tors	500	220,000	20	7.67	293

As it becomes apparent from Table 1, a cantilever-based AFM offers high measurement sensitivity and permits advanced multimodal or multifrequency operation modes. Moreover, cantilevers with a wide range of stiffnesses, resonance frequencies, and tips are available, allowing for the selection of a cantilever that is best suited to a certain measurement situation.

### Force gradient noise and measurement bandwidths

**Figure 9:**
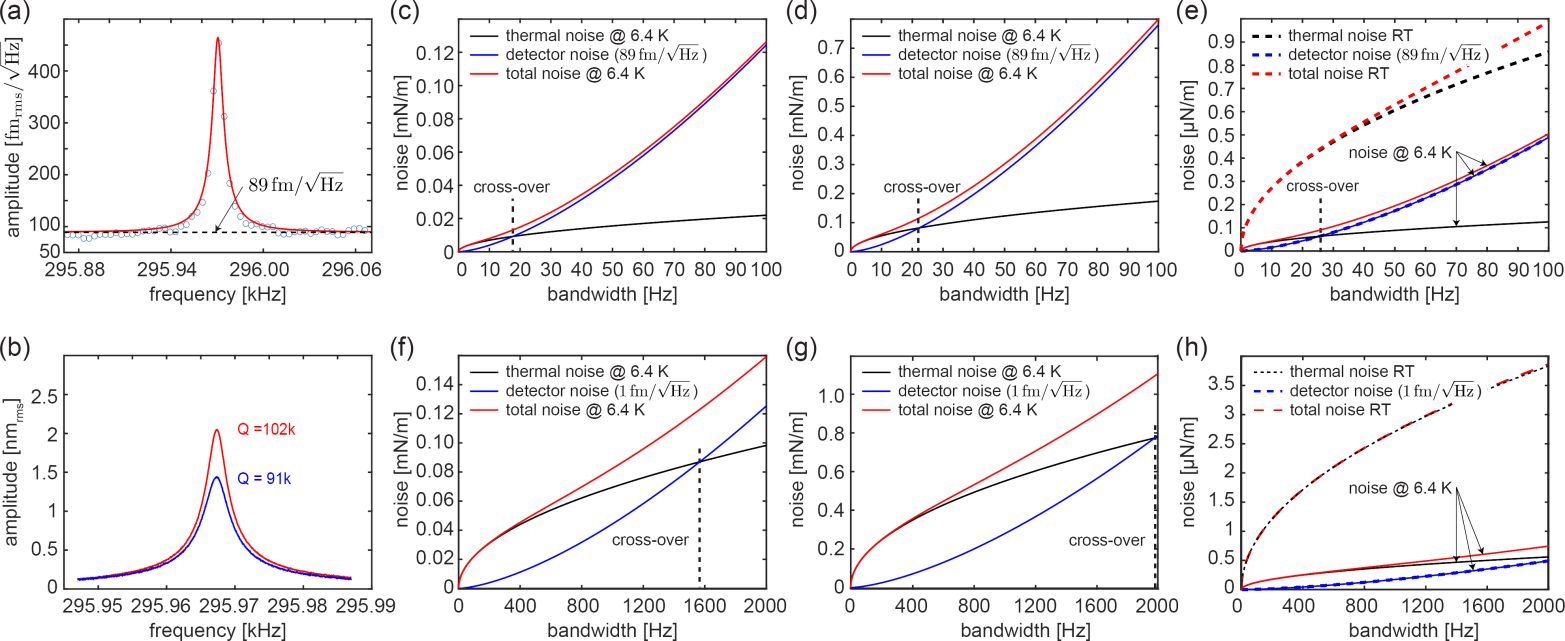
(a) Narrow band thermal noise spectrum of a NCHPt cantilever with length *L* = 125 μm and width *w* = 30 μm around the cantilever first mode flexural resonance. The fitted resonance frequency and interferometer noise floor are *f*_0_ = 295.95 kHz, and 89 fm/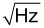, respectively. (b) The measured quality factors on the two interferometer slopes are *Q*_damp_ = 91,000 and *Q*_exc_ = 102,000. (c, d) Force derivative thermal, detector, and total noise in mN/m for the first and second flexural oscillation mode at *T* = 6.4 K, an oscillation amplitude *A* = 100 pm, and a detector noise floor of 89 fm/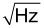, where *k*_1_ = 25.2 N/m, *k*_2_ = 1005 N/m, *f*_1_ = 295.95 kHz, *f*_2_ = 1865 kHz, *Q*_1_ = 100,000, and *Q*_2_ = 10,000. (e) Noise data (here in μN/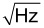) for *T* = 6.4 (solid lines) and *T* = 300 K (dashed lines) for the first flexural mode of an MFM cantilever [29] with a first mode resonance frequency *f*_1_ = 51 kHz, first mode stiffness of 0.86 N/m, an rms oscillation amplitude of *A* = 5 nm, and a first mode quality factor *Q* = 242,000. At higher bandwidths, that is, at 18 Hz (first mode), 22 Hz (second mode), and 25 Hz (MFM cantilever at *T* = 6.4 K), the detector noise becomes the dominant noise source. Panels (f)–(h) display the noise results for bandwidths up to 2000 Hz extrapolated from panels (c)–(e) for a detector noise floor improved to 1 fm/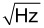 as for example reached by [46] and [47] with different types of fiber-optical Fabry–Perót interferometers.

Figure 9a shows thermal noise data, measured at 6.4 K, of a Nanosensors PPP-NCHPt cantilever with *L* = 125 μm, *w* = 30 μm, and a measured first mode resonance frequency of *f*_0_ = 295.97 kHz, together with the fitted resonance curve and the detector noise of our currently implemented interferometer (which is 89 fm/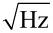 for the non-coated, cleaved fiber end used here). Note that at such laser powers, the cantilever quality factor is increased or decreased by photothermal effects such that two different quality factors are measured for the interferometer working points on the rising and the falling slopes of the interferometer signal [66–68]. Figure 9b displays the two different resonance curves with an enhanced (red curve) and attenuated quality factor (blue curve) measured at a lower laser power than the resonance curve displayed in Figure 9a with the quality factor further attenuated by the higher laser power down to a value of 91,000, as obtained from the fit of the resonance curve. The quality factor relevant for the thermodynamic cantilever noise would be obtained at even lower laser powers than that used to measure the resonance curves displayed in Figure 9b and can be approximated by the mean of the two quality factors, that is, *Q*_1_ = 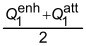 ≈ 100,000. Note that the quality factor of the second flexural mode is not noticeably influenced by the interferometer operation point, but is typically considerably lower than *Q*_1_ (*Q*_2_ ≈ 10,000). We attribute this to energy dissipation arising by instabilities of the atomic positions of atoms inside the grain boundaries [69] of the rather thick metallic coating applied to the tip side of the cantilever. Note that a nominally 20 nm thick Pt coating is required to permit tunneling, but the coating thickness along the cantilever could presumably be minimized using masking procedures similar to those used for the coating of high-quality factor cantilevers for magnetic force microscopy [29]. In future work, much thinner coating thicknesses could be used, or the coating could be applied to the cantilever side to reduce energy dissipation processes arising from the grain boundaries of the polycrystalline coating.

In order to obtain the properties of the cantilever used for our experiments, we use the measured first flexural mode resonance frequency *f*_1_ = 295.95 kHz (Figure 9a), length *L* = 125 μm and width *w* = 30 μm, given by the manufacturer, and Equation 8 to calculate its thickness *t* = 3.352 μm.

Using Equation 7, Equation 5, and Equation 6, the force constant *c*_L_ = 24.4 N/m, the first flexural mode stiffness *k*_1_ = 25.2 N/m, the second flexural mode stiffness *k*_2_ = 1005 N/m, and the second flexural mode resonance frequency *f*_2_ = 1865 kHz were obtained. Note that the second mode resonance frequency calculated from Equation 6 typically differs from the measured second mode resonance frequency by only a few percent. The noise of the interferometer deflection measurement *n*_eq_ = 89 fm/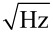 was obtained from fitting the first flexural mode thermal noise spectrum.

Figure 9c,d shows the dependence of the force derivative noise on measurement bandwidth for the first and second flexural modes, respectively, for a rms oscillation amplitude of 100 pm and quality factors *Q*_1_ = 100,000 and *Q*_2_ = 10,000. The measurement sensitivity of the first and second flexural cantilever mode are both limited by thermal noise for measurement bandwidths smaller than 18 and 22 Hz, respectively, and by deflection noise for larger bandwidths. However, for bandwidths up to 100 Hz, the noise remains below 1 mN/m even for the second flexural mode and below 0.1 mN/m for bandwidths smaller than about 22 Hz, as typically used in tuning fork AFM experiments. Sensitivities about one order of magnitude better are, then, obtained in the first cantilever oscillation mode. Note that these values are obtained for a non-optimized interferometer with a noise floor of 89 fm/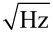 (Figure 9a), clearly demonstrating the superior performance possible with cantilever-based AFM.

For comparison, the dependence of the minimally measurable force derivatives for an MFM cantilever [29] with *f*_1_ = 51.002 kHz, *k*_1_ = 0.86 N/m, and *Q*_1_ = 241,908 obtained at room temperature (solid lines) and 6.4 K (dashed lines) are displayed in Figure 9e for a rms oscillation amplitude of 5 nm (as typically used for MFM [29]). The sensitivity of the softer MFM cantilever (operated at a 50 times larger oscillation amplitude compared to the one used in the second flexural mode) is considerably higher than that of the hard cantilever (note that the scale is given in μN/m instead of mN/m) and not limited by detector noise at room temperature. Such an extremely high force derivative sensitivity is key for MFM experiments with high spatial resolution (and also to minimize the influence of the tip stray field on the sample by employing low magnetic moment tips). In addition, such a sensitivity is also useful for mapping other small forces, such as weak electrostatic, van der Waals, or Casimir forces, highlighting the advantages arising from using cantilevers with optimized force constant for a particular type of tip–sample interaction. At 6.4 K the total noise of the MFM cantilever is again limited by detector noise for bandwidths above 50 Hz. The noise of the deflection sensor employed here is clearly relevant for measurements performed at higher bandwidths at low temperatures for all types of cantilevers. The best interferometer optical sensors have been reported to reach measurement sensitivities of better than 1 fm/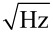 [46–47], a sensitivity not achieved here for our interferometer, which still employs an uncoated fiber end. The sensitivities that could be obtained with such improved interferometer setups are displayed in Figure 9f–h for measurement bandwidths up to 2 kHz. Clearly, the deflection sensor noise does no longer limit the minimally detectable force derivative for bandwidths up to and beyond 1 kHz. Such high measurement bandwidths can for example, be used to measure with high speed a large-scale image showing atomic steps of the Au(111) surface with thin NaCl islands on top (see section “Results and Discussion”).

There is a third noise source, namely the oscillator noise given by Equation 3, which is, however, relevant only for low-quality factor conditions [59]. An experimental evaluation of the measured frequency shift noise revealed that it depends as 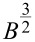 on the bandwidth *B*, confirming that the relevant noise source with our current interferometer sensor is the deflection noise and that the oscillator noise remains negligible (as expected for high-quality factor conditions). Consequently, the high resonance frequency-to-stiffness ratio of microfabricated cantilevers is highly advantageous for AFM measurements with the highest sensitivity or for more rapid scanning, requiring larger measurement bandwidths (see Table 1).

### STM noise spectrum and tip–sample gap stability measurements

**Figure 10:**
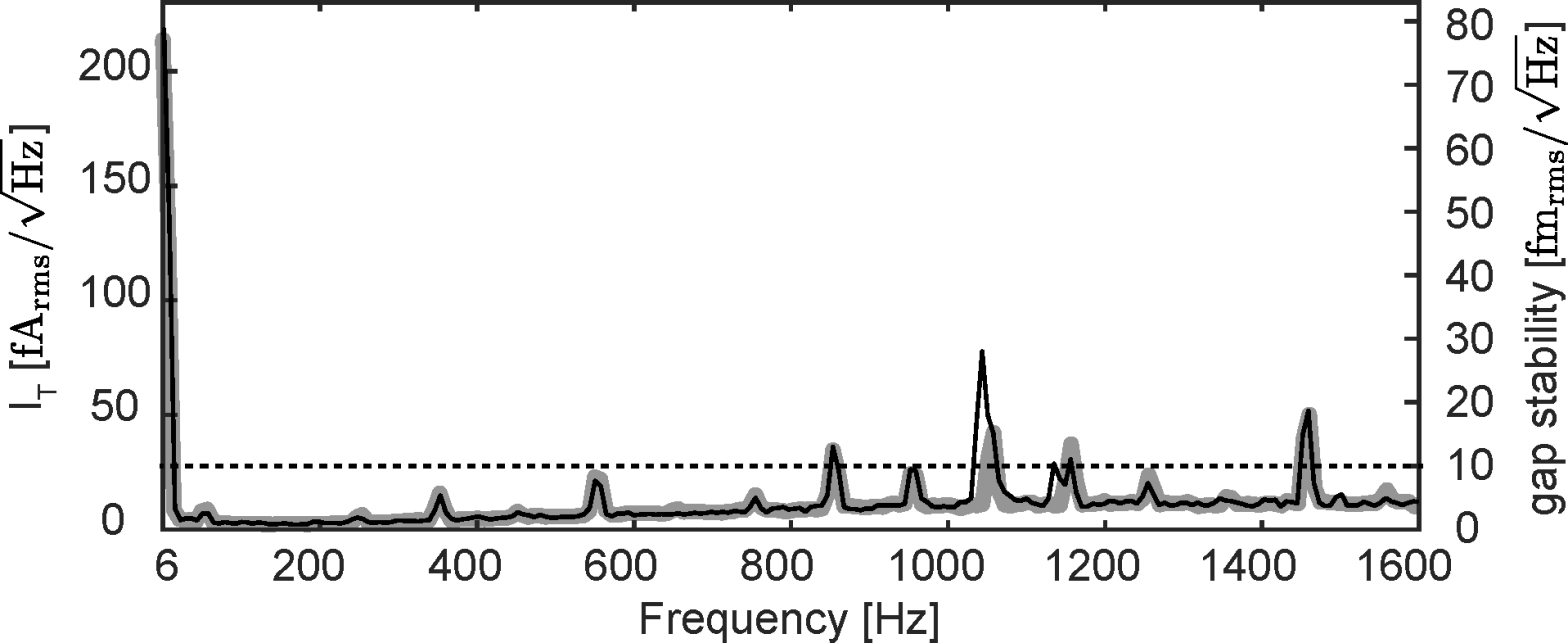
The current noise spectral density with the tip retracted from and approached to the surface of an electrically conducting sample for measurement bandwidths of 0 to 1600 Hz. The current noise spectral density with the retracted tip is displayed as a wide gray curve with the current noise on the left vertical scale. The current noise spectral density with the approached tip is displayed as a solid black line with the tunneling current noise on the left vertical scale and with a converted noise of the tip–sample gap stability on the right vertical scale. The dashed horizontal black line indicates a noise level of 10 fm/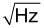.

A scanning probe microscopy tool designed for the acquisition of data with atomic resolution requires a tip–sample gap stability that is, in the best case, better than 1 pm. A convenient method to test the gap stability is to measure the current noise while tunneling on a conducting sample. Figure 10 displays the current noise spectrum up to 1600 Hz for the tip retracted from the surface (wide gray line) and for the tip approached to the surface (thin black line) such that a tunnel current of 20 pA is obtained with a bias of 200 mV, respectively. The noise spectrum (left vertical scale) recorded with the tip retracted from the surface contains a few peaks, which we attribute to the tribo-electric effect [70] and, consequently, tribo-electric currents arising from mechanical vibrations of the cables running along the cryostat. However, all peaks remain smaller than 45 fA_rms_/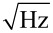.

**Figure 11:**
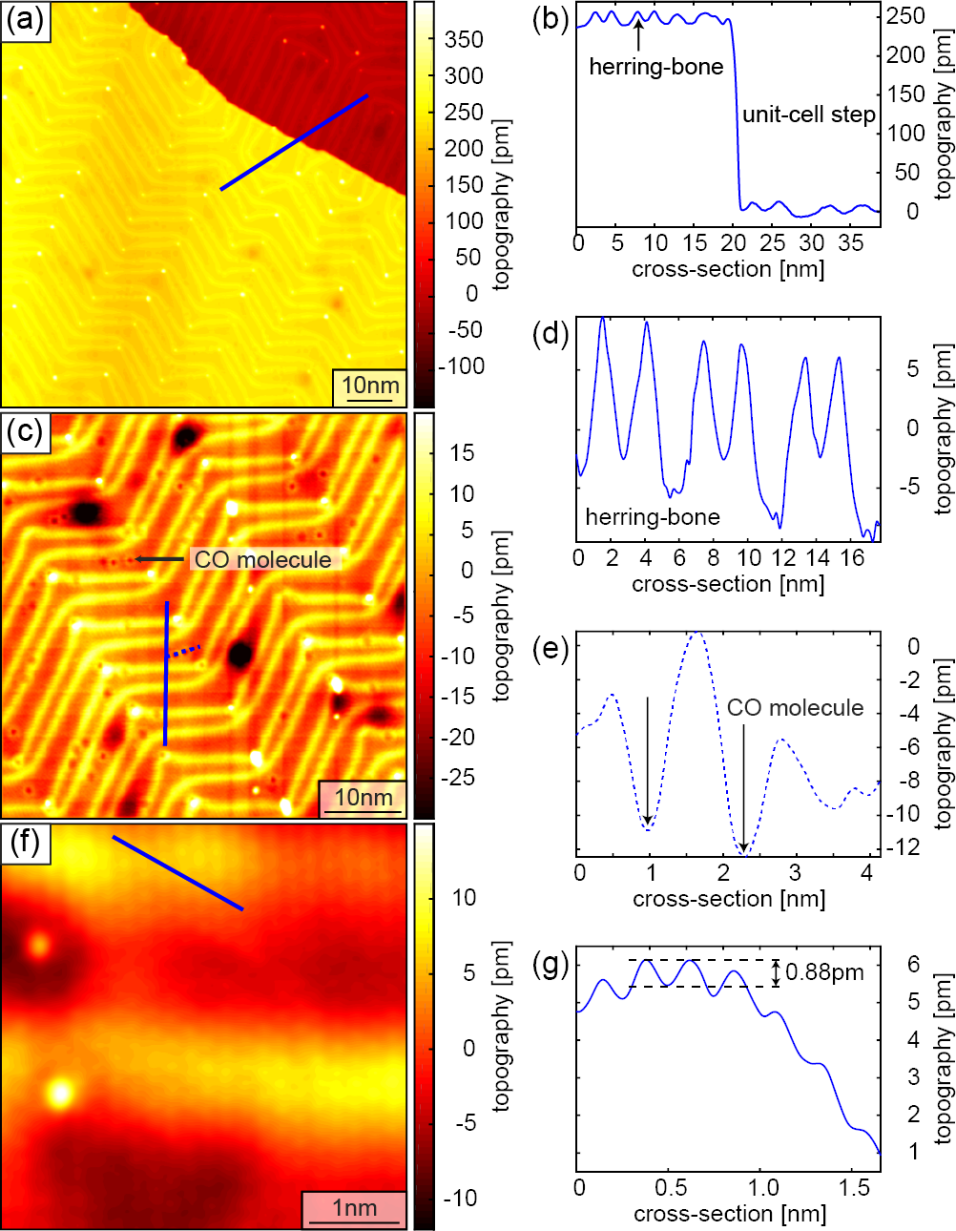
(a, c) STM results on Au(111) obtained at 600 mV and 20 pA. (b, d, e) Cross sections taken at the locations of the solid blue lines in panels (a) and (c) and the dashed blue line in panel (c). (f) Atomic-resolution image acquired at 5 mV and 30 pA. The cross section (g) taken at the location of the blue line in panel (f) shows an atomic corrugation of only 0.88 pm.

If the tip is tunneling, the background noise and most peaks remain unchanged, apart from the peak at 1.05 kHz, which becomes noticeably larger, that is, it doubles from about 40 to 80 fA/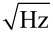. We attribute this increased noise to the thermal noise of the scan piezo that has its first resonance in this frequency range for a Au single crystal sample mounted on a button heater sample holder (Figure 7a–c). Using previously measured tunneling current-versus-sample z-displacement data (not shown), the tunneling current noise data (solid black line in Figure 10 and left vertical axis) can be converted into displacement noise or noise of the tip–sample gap stability (displayed by the right vertical scale in Figure 10). The largest noise at about 1.05 kHz, then, is about 35 fm_rms_/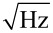. The average noise for the whole spectrum remains below about 10 fm_rms_/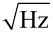 (dashed horizontal black line in Figure 10). Consequently, the integrated rms noise up to a bandwidth of 1600 Hz remains smaller than 400 fm, which permits measurements of sub-pm corrugations as observed for the atomic resolution image on Au(111) performed with an CO-functionalized tunneling tip at a tunnel current setpoint of 30 pA and a bias of 5 mV (see below in Figure 11f).

## Results and Discussion

### STM Measurements

Figure 11a,b shows an STM image and the cross section (taken at the location of the blue line in panel (a)), respectively, of a Au(111) surface acquired at 600 mV and 20 pA. A step and the herringbone structure are well visible.

Figure 11c,d shows a smaller scan area and a cross section acquired on one terrace. Some CO was dosed onto the surface for a successive tip functionalization. The CO molecules appear as dark spots in the image (black arrow). The cross section from Figure 11e taken at the location of the blue dashed line in Figure 11c shows that the CO molecules appear as about 8–10 pm deep depressions. Figure 11f then shows a smaller image acquired at 5 mV and 30 pA, where the herringbone structure is visible together with the atoms. We attribute the extremely small atomic corrugation of less than 1 pm (Figure 11g), to the relatively low current setpoint and to the CO functionalized tip. Nevertheless, corrugations of less than 1 pm can be detected, confirming the excellent tip–sample gap stability of our instrument, compatible with that assessed from the tunnel current noise analysis (Figure 10).

### Rapid scanning and atomic resolution

As discussed in section “Relevant AFM noise sources” and summarized in Table 1, microfabricated cantilevers have a small stiffness-to-resonance frequency ratio, which improves the force derivative sensitivity substantially. Atomic-resolution imaging with AFM is conveniently performed with oscillation amplitudes that are comparable to the decay length of the short-range inter-atomic forces [71]. A stable operation of the PLL with such small oscillation amplitudes requires a cantilever stiffness of a few hundred newtons per meter, such that sufficient energy is stored in the cantilever oscillation [64], that is,


[11]

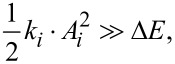



where *k_i_* and *A_i_* are the cantilever stiffness and oscillation amplitude, respectively, of the oscillation mode *i*. Δ*E* is a typical energy loss that can stochastically occur, for example, if the position of an atom within the tip–sample force field becomes instable [63,72]. Such stochastic energy loss processes lead to sudden changes of the phase, which cause the PLL to unlock and, consequently, to a crash of the z-feedback, which is set up to keep the frequency shift constant.

For oscillation amplitudes below 100 pm, Equation 11 reveals that a stiffness above 100 N/m is required for Δ*E* ≈ 1 eV. According to Equation 5, such a cantilever stiffness is conveniently obtained with the second flexural oscillation mode of a cantilever with a first mode stiffness larger than about 10 N/m. Operated in its first flexural mode, such a cantilever then obtains a force derivative sensitivity of better than 0.12 mN/m for a bandwidth of 100 Hz (Figure 9c). Increasing the first mode oscillation amplitude to 2 nm then provides such a sub-mN/m sensitivity even for PLL bandwidths of 2 kHz. These high bandwidths, therefore, permit the rapid scanning of large sample areas, which is convenient for finding a specific are of interest, for example, on a device that will later be scanned with atomic resolution.

Here, we thermally evaporate sub-monolayer NaCl onto a Au(111) surface to obtain a sample surface with different step heights, making large-scale AFM imaging with higher scan rates challenging. The contact potential on Au was compensated by application of a bias of 828 mV. To acquire AFM overview images and, subsequently, atomic resolution images at selected surface locations, including lateral force measurements, we advantageously used the different oscillation modes of a commercial 40 N/m cantilever with first and second flexural, and first torsional mode resonance frequencies of 289, 1829, and 2178 kHz, respectively.

**Figure 12:**
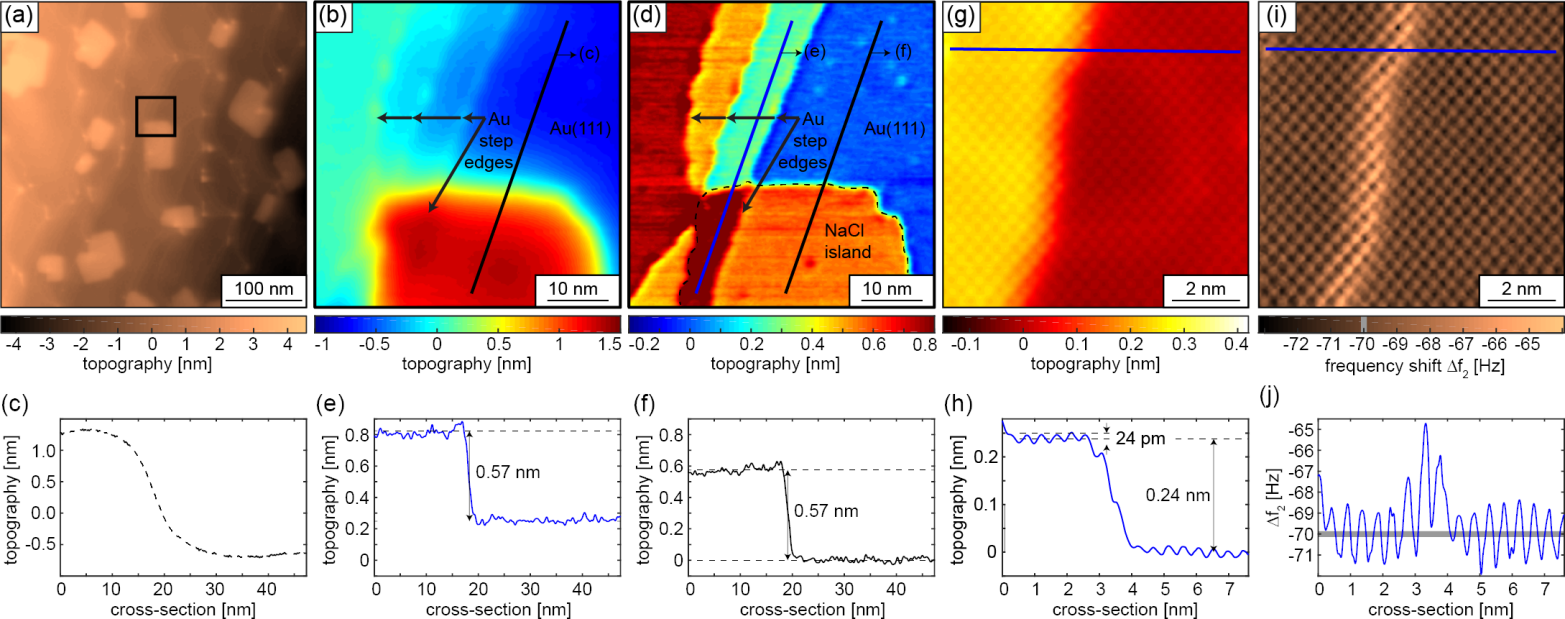
AFM data: (a) 400 × 400 nm^2^ image of NaCl islands on a Au(111) surface scanned with the cantilever operated in its first flexural oscillation mode with an amplitude *A*_f1_*_,_*_rms_ = 2 nm and a small negative frequency shift setpoint Δ*f*_f1_ = −15 Hz, permitting image acquisition at a relatively large tip–sample distance for rapid overview scanning. (b, d) Smaller-scale images acquired in the first and second cantilever oscillation mode operated with amplitudes *A*_f1_*_,_*_rms_ = 2 nm and *A*_f2_*_,_*_rms_ = 100 pm, respectively, at the location of the black square in panel (a), with negative frequency shift setpoints for the first and second flexural mode of Δ*f*_f1_*_,_*_f2_ = −15 Hz. Note that the NaCl islands (enclosed by the dashed line in panel (d)) runs over the lower Au(111) step edge. (c) Cross section taken at the location of the black line in panel (b). (e, f) Cross sections taken at the location of the blue and black lines in panel (d), respectively. (g) Atomic-resolution image and corresponding cross section (h) of the NaCl islands running over the Au(111) step edge measured with the second flexural mode with an oscillation amplitude *A*_f2_*_,_*_rms_ = 100 pm and Δ*f*_f2_ = −70 Hz. (i) Frequency shift error image and corresponding cross section (j).

Figure 12a displays a 400 × 400 nm^2^ AFM image of NaCl islands on a Au(111) surface scanned at 500 ms per line with 256 pixels. A PLL bandwidth of 500 Hz was used for to keep a frequency shift constant at −15 Hz.

Figure 12b shows a zoomed AFM scan at the location of the black square in Figure 12a. Note that the step edge (see cross section displayed in Figure 12c) appears very rounded and the step height is much higher than that expected for two monolayers of NaCl. These observations can be attributed to the relatively large first mode oscillation amplitude (2 nm) and the small negative frequency shift setpoint, such that the frequency shift predominately arises from longer-ranged van der Waals and electrostatic forces and, consequently, a constant frequency shift image does not reflect the true sample topography.

An AFM image acquired at the same location, but using the second flexural mode with an oscillation amplitude of 100 pm, again for a frequency shift setpoint of −15 Hz, is displayed in Figure 12d with the cross section taken at the blue and black lines depicted in Figure 12e,f. The comparison of the step heights of the two cross sections reveals that the NaCl island grows over a unit cell step of the Au(111) surface. The frequency shift Δ*f_i_* measured by an AFM operated in oscillation mode *i* is given by 
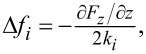
 where ⟨∂*F_z_*/∂*z*⟩ is the interaction force gradient averaged over the oscillation path of the tip [73]. The second flexural oscillation mode of the cantilever has an about 40 times higher modal stiffness (Equation 5) of the first flexural mode. The tip–sample interaction force gradient averaged over the oscillation path of the tip is correspondingly larger. Keeping the same frequency-shift setpoint of −15 Hz as in the first mode, consequently, leads to a reduction of the tip–sample distance. Moreover, because the oscillation amplitude is reduced from 2 to 0.1 nm, the contribution of the short-range force to the frequency shift is considerably larger [12]. Hence, changes of the (long-range) electrostatic force arising from local contact potential variations have a reduced effect on the frequency shift and, thus, on the measured topography. Consequently, the edge of the NaCl island appears much sharper than in the image in Figure 12b acquired with the first flexural oscillation mode and the observed step height of about 0.57 nm; this value corresponds well to the unit cell lattice constant of NaCl of 0.538 nm, that is, to two monolayers of NaCl [74].

For atomic-resolution imaging, the tip was CO-functionalized on the Au surface, which changes the contact potential substantially such that the bias had to be reduced from 828 to −28 mV. Figure 12g was acquired using a more negative frequency shift kept constant at −70 Hz on a 9 × 9 nm^2^ selected inside the NaCl islands covering a Au(111) step edge. As visible in the cross section displayed in Figure 12h, the observed step height of 0.24 nm corresponds to that of a monolayer step of the Au(111) surface, and the atomic-scale periodicity is about 0.5 nm, less than the bulk lattice constant of 0.538 nm, as expected for a thin 2D NaCl sheet [74]. Figure 12i and Figure 12j show the frequency shift (error) image and cross section, respectively. The atomic-scale corrugation of 24 pm (Figure 12h) leads to a frequency shift error of ±1 Hz around the frequency shift setpoint of −70 Hz, while the Au step leads to a lager frequency shift error of about −5 Hz (Figure 12j).

**Figure 13:**
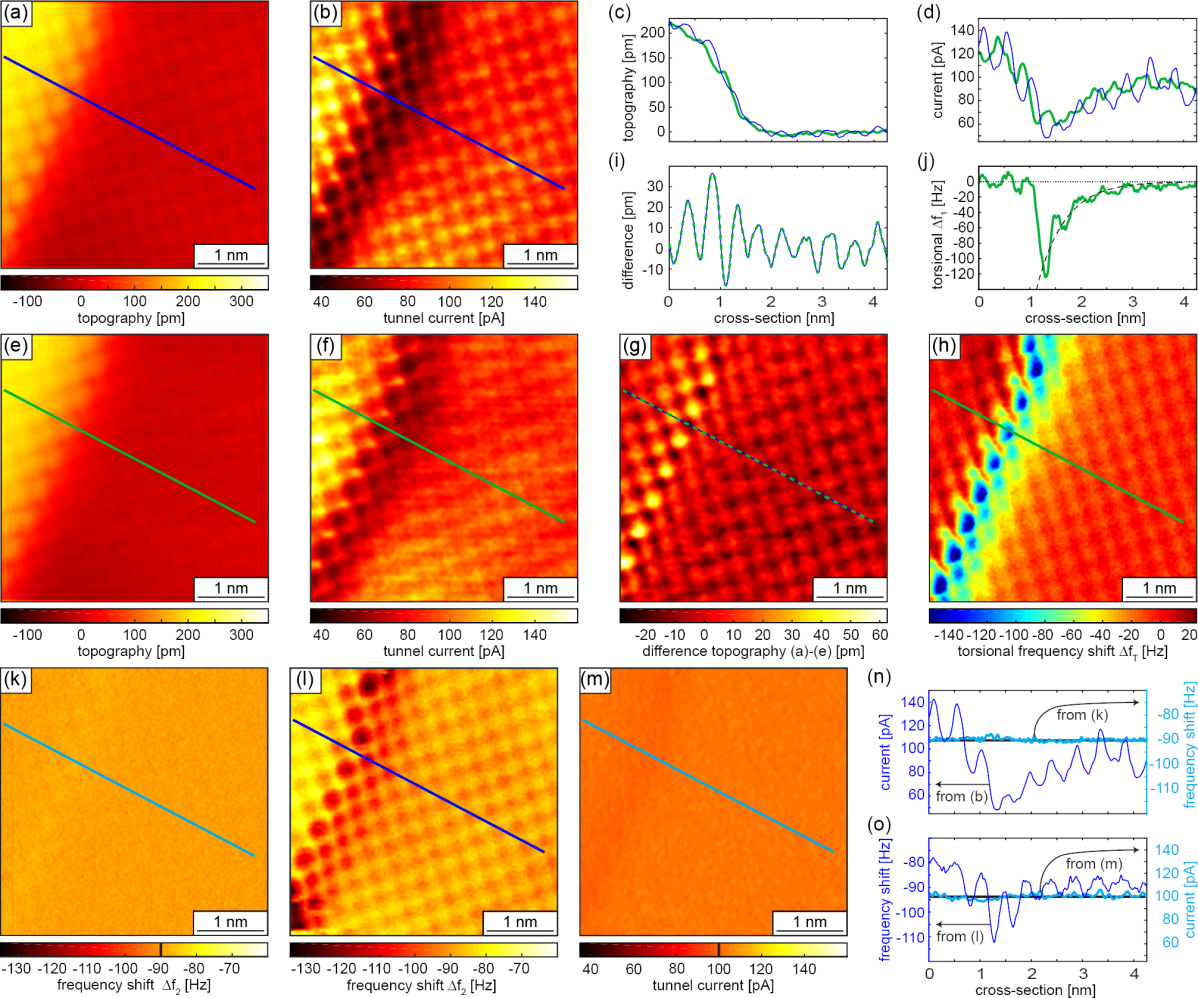
Multichannel and multimodal AFM results obtained on a NaCl island running over a Au(111) step edge. (a) Topography and (b) tunnel current images obtained with the second flexural mode frequency shift Δ*f*_f2_ = −90 Hz and a second mode oscillation amplitude *A*_f2_*_,_*_rms_ = 100 pm. The blue lines in panels (c) and (d) represent the cross sections taken at the location of the blue lines in (a) and (b), respectively. Panels (e) and (f) show the same quantities as panels (a) and (b) but with the cantilever oscillated simultaneously in its first torsional mode with a torsional mode amplitude *A*_t1_*_,_*_rms_ = 60 pm to obtain the torsional mode frequency shift image Δ*f*_t1_(*x*,*y*) displayed in panel (h). A large lateral attractive force is observed when the tip is approached to the step edge from the lower terrace side. See green cross section in panel (j). Because of the additional lateral tip oscillation, the topographical corrugation in panel (e) is slightly reduced compared to that in panel (a). Compare also the topography and tunnel current cross-sections, that is, the green and blue lines in panels (c) and (d), respectively. The reduction of the topographical corrugation is particularly pronounced at the step edge as visible in the difference data displayed in panel (g) calculated by subtracting the data shown in panel (a) from that displayed in panel (e). (i) The green dashed cross section in panel (g). (k) The frequency shift error observed during the constant frequency shift imaging used for the data displayed in panels (a) and (b). Alternatively, the tunnel current can be kept constant. Then the frequency shift shows an atomic-scale contrast (l). The corresponding tunnel current error image is displayed in panel (m). Panels (n) and (o) show the tunnel current and frequency shift variations along the cross sections indicated by the lines in panels (b) and (l), respectively, while the frequency shift or tunnel current is kept constant (pale blue lines in panels (n) and (o)).

Apart from using different flexural cantilever oscillation modes for rapid large-scale and local atomic-resolution imaging, the cantilever can also be oscillated in its torsional modes, permitting the measurement of lateral forces or multimodal operation of flexural and torsional oscillation modes [33–34,75]. Here, we demonstrate that positioning the fiber-end of the interferometric deflection sensor outside the cantilever long axis, close to its edges (Figure 8e), the torsional cantilever oscillation mode can be measured simultaneously with the flexural ones (Figure 8d,f). Similar to the work of Kawai et al. [35], we operate the z-feedback on the second flexural mode frequency to control the tip–sample distance, while simultaneously imaging the frequency shift of the first torsional mode to map the lateral tip–sample force derivative (along the torsional oscillation axis of the tip), or alternatively use the tunnel current for the z-feedback. Figure 13a displays a 4 × 4 nm^2^ topography image of a NaCl island overgrowing a step edge of the Au(111) surface. The data was acquired with a second flexural mode frequency shift Δ*f*_f2_ kept constant at −90 Hz and an oscillation amplitude *A*_f2_*_,_*_rms_ = 100 pm, while Figure 13b shows the simultaneously measured tunnel current image obtained for a bias of 100 mV. The blue lines in Figure 13c,d display cross-sectional data of the topography (Figure 13a) and tunnel current (Figure 13b) images, respectively. Interestingly, the current drops to a minimum of about 55 pA when the tip scans from the upper to the lower terrace, indicating that the tip is a bit farther away from the surface in the vicinity of the step edge. This is because a part of the mesoscopic tip is still located above the upper terrace contributing to an increased negative Δ*f*_f2_. Only if the tip moves farther away from the step edge, the average tunnel current and the tunnel current corrugation level recover to the value measured away from the step edge on the upper terrace. From larger-scale images (not shown) we can conclude that size of the tip apex must have a diameter smaller than about 15 nm. If the cantilever is additionally driven in the first torsional mode with an amplitude *A*_t1_*_,_*_rms_ = 60 pm, the atomic resolution in the topography image from Figure 13e and the cross section displayed as green line in Figure 13c is still visible, but reduced considerably. The difference data displayed in Figure 13g and the corresponding cross-sectional data in Figure 13i reveal that the contrast reduction is most significant at the step edge. Atomic resolution was also obtained in the torsional frequency shift Δ*f*_t1_ data shown in Figure 13h.

As already observed by Kawai et al. [35], a strong negative torsional frequency shift appears as the tip approaches to the step from the lower terrace side, which must arise from a rather strong attractive lateral force towards the step edge. The dashed line in Figure 13j shows the result of a fit in the cross section interval [1.26 nm, 4.255 nm] of two exponential decay functions with wavelengths fixed at λ_1_ = 3.6 nm and λ_2_ = 0.5 nm, corresponding to the Fermi wavelength of the Au(111) free electron-like surface state [76], and approximately the NaCl ion periodicity, respectively. This indicates that the lateral force may arise from a charge on the step edge of Au(111) and a contribution from the periodic charges of the ionic lattice. On the upper side, the atomic corrugation is also visible but, in contrast to Kawai et al., no overall attractive force (negative torsional frequency shift) is visible.

Atomic-resolution images can be obtained with different z-feedback input signals. Figure 13b shows the tunnel current data obtained with the second mode flexural frequency Δ*f*_f2_ = −90 Hz. The Δ*f*_f2_ error signal data shown in Figure 13k reveals that the frequency shift is kept within about ±1 Hz. Correspondingly, Figure 13l shows the second mode flexural frequency data if the tunnel current is kept at 100 pA (Figure 13m is the corresponding current error data). Figure 13n and Figure 13o show cross-sectional data for the two feedback setups. These cross sections again confirm that atomic resolution data can be obtained either in tunnel current when the frequency shift is kept constant at −90 Hz or in the frequency shift, if the tunnel current is kept constant at 100 pA.

## Conclusion

In this article, we have described design and construction of a cantilever-based low-temperature UHV AFM with sub-picometer gap stability, which enables multimodal and multidimensional AFM operation combined with STM. The use of microfabricated cantilevers requires the implementation of an additional deflection sensor, which increases the complexity of the instrument. However, the low ratio of stiffness to resonance frequency (stemming from the small geometrical dimensions of cantilevers) significantly reduces thermal and deflection noise force derivatives. Because the latter is often the dominating noise source (particularly for tuning fork-based AFM instrumentation), the cantilever-based AFM instrument presented here has a two orders of magnitude increased force derivative sensitivity, permitting high AFM measurement bandwidths, typically of a few hundred hertz, which could be further increased to 2 kHz with improved interferometric detection [46–47]). Further, because a larger variety of cantilevers with a large stiffness range is available, cantilevers optimized for a special experimental task can be used, for example, for magnetic force microscopy with the highest field sensitivity [29] or atomic-resolution work (as shown here). In addition, microfabricated cantilevers permit multimodal operation, for example, for magnetic force microscopy with capacitive tip–sample distance control [32], or the simultaneous mapping of vertical and lateral forces and the tunnel current with atomic-scale resolution as demonstrated here. Future scientific frontiers may require an AFM-based search on the micrometer-scale over device structures including insulating parts, thus, requiring an AFM imaging tool that can accomplish large-area scans using weak van der Waals forces with a relatively large tip–sample distance permitting robust overview scanning.

## References

[1] Kirk M D, Albrecht T R, Quate C F (1988). Rev Sci Instrum.

[2] Metz V, Raanan H, Pieper H, Bosbach D, Ganor J (2005). Geochim Cosmochim Acta.

[3] Schulz F, Maillard J, Kaiser K, Schmitz-Afonso I, Gautier T, Afonso C, Carrasco N, Gross L (2021). Astrophys J, Lett.

[4] Giessibl F J (1995). Science.

[5] Ueyama H, Ohta M, Sugawara Y, Morita S (1995). Jpn J Appl Phys, Part 1.

[6] Kitamura S-i, Iwatsuki M (1995). Jpn J Appl Phys, Part 1.

[7] Sugawara Y, Ohta M, Ueyama H, Morita S (1995). Science.

[8] Gross L, Mohn F, Moll N, Liljeroth P, Meyer G (2009). Science.

[9] Alldritt B, Urtev F, Oinonen N, Aapro M, Kannala J, Liljeroth P, Foster A S (2022). Comput Phys Commun.

[10] Mönig H, Hermoso D R, Díaz Arado O, Todorović M, Timmer A, Schüer S, Langewisch G, Pérez R, Fuchs H (2016). ACS Nano.

[11] Mohn F, Schuler B, Gross L, Meyer G (2013). Appl Phys Lett.

[12] Giessibl F J (2019). Rev Sci Instrum.

[13] Schwenk J, Kim S, Berwanger J, Ghahari F, Walkup D, Slot M R, Le S T, Cullen W G, Blankenship S R, Vranjkovic S (2020). Rev Sci Instrum.

[14] Albers B J, Schwendemann T C, Baykara M Z, Pilet N, Liebmann M, Altman E I, Schwarz U D (2009). Nat Nanotechnol.

[15] Garcia R, Herruzo E T (2012). Nat Nanotechnol.

[16] Platz D, Tholén E A, Pesen D, Haviland D B (2008). Appl Phys Lett.

[17] Li J W, Cleveland J P, Proksch R (2009). Appl Phys Lett.

[18] Dietz C, Herruzo E T, Lozano J R, Garcia R (2011). Nanotechnology.

[19] Forchheimer D, Platz D, Tholén E A, Haviland D B (2012). Phys Rev B.

[20] Forchheimer D, Platz D, Tholén E A, Haviland D B (2013). Appl Phys Lett.

[21] Nievergelt A P, Adams J D, Odermatt P D, Fantner G E (2014). Beilstein J Nanotechnol.

[22] Nievergelt A P, Erickson B W, Hosseini N, Adams J D, Fantner G E (2015). Sci Rep.

[23] Penedo M, Hug H J (2018). Appl Phys Lett.

[24] Braunsmann C, Schäffer T E (2010). Nanotechnology.

[25] Young T J, Monclus M A, Burnett T L, Broughton W R, Ogin S L, Smith P A (2011). Meas Sci Technol.

[26] Collins L, Belianinov A, Proksch R, Zuo T, Zhang Y, Liaw P K, Kalinin S V, Jesse S (2016). Appl Phys Lett.

[27] Kalinin S V, Strelcov E, Belianinov A, Somnath S, Vasudevan R K, Lingerfelt E J, Archibald R K, Chen C, Proksch R, Laanait N (2016). ACS Nano.

[28] Rugar D, Stipe B C, Mamin H J, Yannoni C S, Stowe T D, Yasumura K Y, Kenny T W (2001). Appl Phys A: Mater Sci Process.

[29] Feng Y, Vaghefi P M, Vranjkovic S, Penedo M, Kappenberger P, Schwenk J, Zhao X, Mandru A-O, Hug H J (2022). J Magn Magn Mater.

[30] Schwenk J, Marioni M, Romer S, Joshi N R, Hug H J (2014). Appl Phys Lett.

[31] Schwenk J, Zhao X, Bacani M, Marioni M A, Romer S, Hug H J (2015). Appl Phys Lett.

[32] Zhao X, Schwenk J, Mandru A O, Penedo M, Baćani M, Marioni M A, Hug H J (2018). New J Phys.

[33] Pfeiffer O, Bennewitz R, Baratoff A, Meyer E, Grütter P (2002). Phys Rev B.

[34] Kawai S, Kitamura S-i, Kobayashi D, Kawakatsu H (2005). Appl Phys Lett.

[35] Kawai S, Canova F F, Glatzel T, Hynninen T, Meyer E, Foster A S (2012). Phys Rev Lett.

[36] Kawai S, Glatzel T, Koch S, Such B, Baratoff A, Meyer E (2010). Phys Rev B.

[37] Kawai S, Pina C M, Bubendorf A, Fessler G, Glatzel T, Gnecco E, Meyer E (2013). Nanotechnology.

[38] Kawai S, Pawlak R, Glatzel T, Meyer E (2011). Phys Rev B.

[R39] 39The UHV chambers and cryosystem were fabricated by Createc GmbH as a modified version of their UHV low-temperature STM.

[R40] 40CryoVac GmbH & Co. KG, D-53842 Troisdorf, Germany.

[R41] 41PH BRONZE 5 CDA 510 A, California Fine Wire Co., CA 93433, USA.

[42] Meyer G, Amer N M (1988). Appl Phys Lett.

[43] Rugar D, Mamin H J, Guethner P (1989). Appl Phys Lett.

[44] Moser A, Hug H J, Jung T, Schwarz U D, Guntherodt H-J (1993). Meas Sci Technol.

[45] Hug H J, Stiefel B, van Schendel P J A, Moser A, Martin S, Güntherodt H-J (1999). Rev Sci Instrum.

[46] Hoogenboom B W, Frederix P L T M, Yang J L, Martin S, Pellmont Y, Steinacher M, Zäch S, Langenbach E, Heimbeck H-J, Engel A (2005). Appl Phys Lett.

[47] Karc Ö, Çelik Ü, Oral A (2020). Rev Sci Instrum.

[48] Pan S H, Hudson E W, Davis J C (1999). Rev Sci Instrum.

[49] Hug H J, Stiefel B, van Schendel P J A, Moser A, Hofer R, Martin S, Güntherodt H-J, Porthun S, Abelmann L, Lodder J C (1998). J Appl Phys.

[R50] 50Songhan Plastic Technology Co.,Ltd., 200120 Shanghai, China.

[R51] 51SS coaxial cable, CC-SS-100, Lake Shore Cryotronics, Inc., Woburn, MA 01801, USA.

[R52] 522451 DAFLON Microminiature PTFE Coated Hook-Up Wire,Daburn Electronics & Cable., Dover, NJ 07801, USA.

[53] Miyahara Y, Griffin H, Roy-Gobeil A, Belyansky R, Bergeron H, Bustamante J, Grutter P (2020). EPJ Tech Instrum.

[R54] 54Custom-made by SFK Schulz GmbH, 12555 Berlin, Germany.

[R55] 55Thorlabs GmbH (Lübeck), 23562 Lübeck, Germany.

[56] Kawai S, Glatzel T, Such B, Koch S, Baratoff A, Meyer E (2012). Phys Rev B.

[57] Reinstaedtler M, Rabe U, Scherer V, Turner J A, Arnold W (2003). Surf Sci.

[58] Kawai S, Glatzel T, Hug H-J, Meyer E (2010). Nanotechnology.

[59] Kobayashi K, Yamada H, Matsushige K (2009). Rev Sci Instrum.

[60] Butt H-J, Jaschke M (1995). Nanotechnology.

[61] Morita S, Giessibl F J, Meyer E (2015). Noncontact Atomic Force Microscopy: Volume 3.

[62] Lübbe J, Tröger L, Torbrügge S, Bechstein R, Richter C, Kühnle A, Reichling M (2010). Meas Sci Technol.

[63] Ghasemi S A, Goedecker S, Baratoff A, Lenosky T, Meyer E, Hug H J (2008). Phys Rev Lett.

[64] Giessibl F J, Hembacher S, Herz M, Schiller C, Mannhart J (2004). Nanotechnology.

[65] Kirpal D, Qiu J, Pürckhauer K, Weymouth A J, Metz M, Giessibl F J (2021). Rev Sci Instrum.

[66] Hölscher H, Milde P, Zerweck U, Eng L M, Hoffmann R (2009). Appl Phys Lett.

[67] Cohadon P F, Heidmann A, Pinard M (1999). Phys Rev Lett.

[68] Metzger C, Ludwig M, Neuenhahn C, Ortlieb A, Favero I, Karrai K, Marquardt F (2008). Phys Rev Lett.

[69] Adiga V P, Sumant A V, Suresh S, Gudeman C, Auciello O, Carlisle J A, Carpick R W (2009). Phys Rev B.

[70] Xu C, Zi Y, Wang A C, Zou H, Dai Y, He X, Wang P, Wang Y-C, Feng P, Li D (2018). Adv Mater (Weinheim, Ger).

[71] Giessibl F J (2003). Rev Mod Phys.

[72] Hoffmann R, Baratoff A, Hug H J, Hidber H R, v. Löhneysen H, Güntherodt H-J (2007). Nanotechnology.

[73] Welker J, Illek E, Giessibl F J (2012). Beilstein J Nanotechnol.

[74] Hebenstreit W, Redinger J, Horozova Z, Schmid M, Podloucky R, Varga P (1999). Surf Sci.

[75] Kawai S, Glatzel T, Koch S, Such B, Baratoff A, Meyer E (2009). Phys Rev Lett.

[76] Sotthewes K, Nijmeijer M, Zandvliet H J W (2021). Phys Rev B.

